# Early-life factors associated with developmental trajectories of internalizing and externalizing symptoms from early childhood through adolescence: Rhea mother—child cohort in Crete, Greece

**DOI:** 10.1007/s00127-026-03103-6

**Published:** 2026-05-11

**Authors:** Katerina Koutra, Chrysi Mouatsou, Katerina Margetaki, Theano Roumeliotaki, Sofia Psoma, Mariza Kampouri, Lida Chatzi

**Affiliations:** 1 Department of Psychology, School of Social Sciences, University of Crete, Gallos Campus 74100, Rethymno, Crete Greece; 2 Clinic of Ρreventive and Social Medicine, Department of Social Medicine, Faculty of Medicine, University of Crete, Heraklion, Crete Greece; 3 Institute of Environmental Medicine, Karolinska Institutet, Stockholm, Sweden; 4 Department of Preventive Medicine, Keck School of Medicine, University of Southern California, Los Angeles, USA

**Keywords:** Internalizing symptoms, Externalizing symptoms, ADHD, Developmental trajectories, Early-life factors

## Abstract

**Purpose:**

The developmental trajectories of internalizing and externalizing symptoms, including ADHD, vary widely among individuals, yet the factors that contribute to these diverse patterns are not fully understood. Our specific objectives are to empirically identify developmental trajectories of internalizing, externalizing, and ADHD symptoms from ages 4 to 15, and to explore how early-life risk factors are linked to these trajectory groups.

**Methods:**

The study included 551 mother–child pairs of the Rhea mother–child cohort in Crete, Greece. Children’s internalizing, externalizing, and ADHD symptoms were evaluated using maternal reports at ages 4 (Strengths and Difficulties Questionnaire, ADHD Test), 6, 11 and 15 years (Child Behavior Checklist, Conners’ Parent Rating Scale-Revised). Group-based trajectory modeling was applied to identify trajectory groups from 4 to 15 years and multinomial logistic regression models were implemented to examine the associations between early-life risk factors and group trajectories.

**Results:**

The analysis revealed four distinct trajectories for each outcome (internalizing, externalizing, and ADHD symptoms): *stable low*, *high-decreasing*, *low-increasing*, and *stable high* symptoms. Furthermore, several early-life sociodemographic and perinatal factors, such as sex, maternal and paternal age, paternal education, maternal smoking during pregnancy, and breastfeeding duration were significantly linked to the development of internalizing, externalizing, and ADHD symptoms.

**Conclusion:**

The study highlights the complex and dynamic nature of emotional and behavioral symptom development and the critical role of early-life determinants in shaping these trajectories. Findings suggest that early identification and intervention are crucial for preventing the persistence of psychopathology into adolescence.

**Supplementary Information:**

The online version contains supplementary material available at https://doi.org/10.1007/s00127-026-03103-6.

## Introduction

Internalizing disorders (e.g., anxiety, depression) and externalizing disorders (e.g., conduct disorder, attention deficit hyperactivity disorder—ADHD) are common in children and adolescents, affecting 10% to 20% [1, 2] of this group. Both types of disorders can emerge in early childhood and affect development if untreated [3]. Internalizing and externalizing disorders in childhood can significantly impact mental health later in life, often leading to persistent psychological challenges and a higher risk of developing mood, anxiety, and behavioral disorders in adulthood [4]. Among externalizing disorders, ADHD is the most prevalent, characterized by early onset and a strong association with long-term outcomes, including academic difficulties, social challenges, and an increased risk of further psychopathology or comorbid conditions [5, 6].

Numerous studies using person-oriented approaches, which focus on individual developmental trajectories rather than population averages, have identified distinct developmental trajectories for internalizing and externalizing symptoms in children, revealing patterns such as persistently high, fluctuating, or remitting symptoms throughout development. For example, internalizing symptom trajectories from toddlerhood to childhood include low, increasing, decreasing, high, and preschool-limited patterns [7, 8], while studies focusing on childhood years have found trajectories such as persistently low, moderate, increasing, decreasing, and stable or rising high symptoms [9, 10]. Similar trends continue into adolescence, with trajectories including low, decreasing, increasing, moderate, and persistently high symptoms [11–14]. For externalizing symptoms, most children follow low symptom trajectories, with smaller subgroups showing increasing, decreasing, or chronic symptoms [10, 12, 15–17]. Across studies, it is consistently found that 11% to 30% of children experience persistently high or increasing emotional and behavioral problems [10].

Sociodemographic factors play a critical role in shaping the developmental trajectories of internalizing and externalizing symptoms in children. Research shows that females are more prone to internalizing disorders, while males are more likely to exhibit externalizing behaviors [18]. Evidence indicates that males and females follow distinct mental health symptom trajectories, underscoring the importance of considering gender differences when conducting assessments and designing interventions [13, 19–22]. Additionally, studies suggest that higher maternal age at childbirth can reduce the risk of emotional and behavioral difficulties in children [23, 24]. While higher parental education generally correlates with better mental health outcomes across development [25–28], its influence on symptom progression remains ambiguous. Some research, such as Tearne et al. [24], suggests that lower maternal education may be linked to worsening mental health issues during adolescence. In comparison, household income appears to be a stronger predictor of symptom trajectories, with children from lower socioeconomic backgrounds more likely to experience persistently high emotional symptoms from ages 6 to 13 [29]. Conversely, higher family income has been identified as a protective factor against adverse mental health outcomes during childhood [30].

Perinatal factors have long been recognized for their significant impact on development, with evidence suggesting that antenatal and early-life adversities, including sociodemographic challenges such as low socioeconomic status and parental educational level, can influence the developing organism and increase the risk of diseases later in life [31]. Low birthweight and preterm birth are associated with heightened emotional symptoms, ADHD, and depression across various developmental stages, from childhood to adulthood [29, 32, 33]. Maternal smoking during pregnancy also elevates the risk of both internalizing and externalizing psychopathology [34–36]. On the other hand, breastfeeding has been found to serve as a protective factor against emotional and behavioral symptoms from childhood through adolescence [37, 38]. The relationship between birth order and mental health remains mixed, with some studies suggesting that firstborn children are more likely to experience internalizing, externalizing, and ADHD problems [39, 40], while others indicate that later-born children may face a greater risk for mental health issues [41].

The increasing prevalence and impact of mental health issues in children and adolescents highlight the critical need to understand the long-term development of internalizing and externalizing disorders from early childhood through adolescence. The lack of comprehensive longitudinal data, especially from diverse cultural and socioeconomic backgrounds, limits our understanding of how these symptoms evolve over time. Early identification of at-risk children is vital, as the early-childhood and adolescent years are crucial for psychological and neurodevelopmental growth, making timely interventions particularly effective. To our knowledge, no studies have explored the association between early-life sociodemographic and perinatal factors and developmental trajectories from childhood to adolescence in Greece. Thus, the present study aims to (1) explore the developmental trajectories of internalizing, externalizing, and ADHD symptoms in a population-based sample of Greek children aged 4 to 15 years, and (2) identify early-life sociodemographic and perinatal factors associated with these developmental trajectories, providing insights into how these factors influence mental health outcomes in this population. ADHD trajectories were analyzed separately from broader externalizing symptoms. Given ADHD’s prevalence, early onset, and its well-documented associations with distinct developmental patterns (i.e., persistence, fluctuation, or remission of symptoms over time) [42], this approach allows for a clearer understanding of ADHD’s distinct contribution to symptom progression, without conflating it with other externalizing behaviors.

## Methods

### Participants

The present study is part of an ongoing population-based mother–child cohort (Rhea Study) in Crete, Greece. Between February 2007 and February 2008, 1,610 pregnant women agreed to participate, resulting in the follow-up of 1,363 singleton pregnancies until delivery. To be eligible for inclusion, participants had to reside in the Heraklion prefecture, be over 16 years old, and had a good understanding of the Greek language. Mothers were followed throughout pregnancy, delivery, and postpartum, while children were assessed during infancy (around 9 and 18 months), early childhood (approximately 4.2 years), mid-childhood (about 6.5 years), pre-adolescence (around 11 years), and adolescence (15 years). At the 15-year follow-up, an in-depth evaluation of maternal and child mental health was conducted as part of the IntExt Trajectories project. A comprehensive overview of the cohort profile, follow-up visits, and the measures utilized can be found in Chatzi et al. [43].

**Fig. 1:**
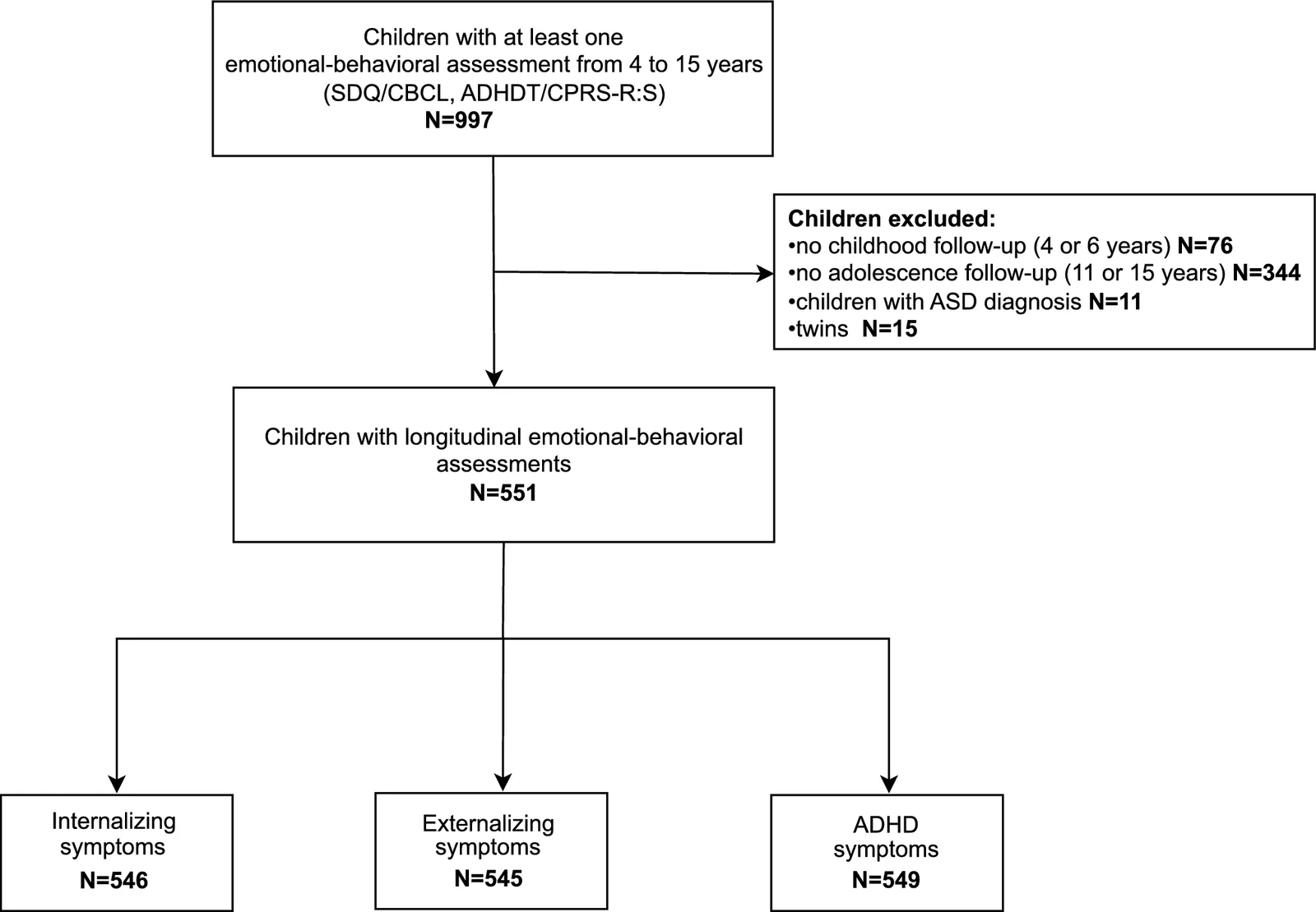
Flowchart of the study population. *Abbreviations*: ADHDT: Attention Deficit Hyperactivity Disorder Test; ASD: Autism Spectrum Disorder; CBCL: Child Behavior Checklist; CPRS-R:S: Conners’ Parent Rating Scale-Revised: Short Form; SDQ; Strengths and Difficulties Questionnaire

To assess emotional and behavioral outcomes over time, data from follow-up assessments conducted at ages 4, 6, 11, and 15 years were utilized. For the longitudinal analysis, we began with a sample of 997 children who had at least one assessment of emotional and behavioral development between the ages of 4 and 15. We established the criterion of having at least two assessments—one during childhood (ages 4 or 6) and another during pre-adolescence/adolescence (ages 11 or 15)—to effectively capture both developmental stages. After excluding twins (n = 15) and children diagnosed with autism spectrum disorder (n = 11), the final sample comprised 551 children (Fig. 1).

The study was conducted in accordance with the principles of the Helsinki Declaration, with all procedures and evaluations approved by the Ethics Committee of the University Hospital of Heraklion (reference number: 96/06.02.2007) and by the Research Ethics Committee of the University of Crete (reference number: 43/16.03.2022) at the most recent follow-up (IntExt Trajectories Project). Written informed consent was obtained from all participants.

### Measures

#### Early-life sociodemographic and perinatal risk factors

Parental sociodemographic characteristics were gathered using questionnaires administered by interviewers. Information about maternal and paternal age (years), parental educational level (low: ≤ 9 years of mandatory schooling, medium: > 9 years of mandatory schooling up to attending post-secondary school education, high: attending or having a university/technical college degree), family origin (both parents Greek, both parents foreign/mixed), parental employment status during pregnancy (unemployed, working), marital status (married, other), parity (nulliparous, multiparous), residential area (urban, rural), and family income was collected during the first trimester of pregnancy. Family income was categorized into tertiles (1st Tertile < 885€, 2nd Tertile 885–1223€, 3rd Tertile 1223 – 2180 €, missing).

Information on perinatal factors, collected through questionnaires and clinical records, included maternal smoking status during pregnancy (non-smokers, early pregnancy smokers, during pregnancy smokers), maternal pre-pregnancy Body Mass Index (BMI; kg/m^2^), child sex (male, female), mode of delivery (vaginal, caesarean section), preterm birth (yes, no), birth order (first, second, third or more), birth weight (grams), birth length (centimeters), birth head circumference (centimeters), gestational age (weeks), admission to the intensive care unit after birth (yes, no), breastfeeding duration (months). Gestational age and anthropometric measurements at birth were collected from clinical records at delivery. Gestational age was calculated based on the interval between the last menstrual period and the delivery date. If the menstrual-based estimate of gestational age differed by seven or more days from the first-trimester ultrasound measurement, a quadratic regression formula linking crown-rump length to gestational age was used [44]. Information on breastfeeding duration (in months) was collected at 9 months postpartum.

#### Children’s internalizing and externalizing symptoms

Mothers assessed their children’s internalizing and externalizing symptoms using the Strengths and Difficulties Questionnaire (SDQ) [45] at age 4. The SDQ is a concise tool designed to assess emotional, behavioral, and relational strengths and difficulties in children aged 4 to 17 years. The parent version consists of 25 items rated on a 3-point Likert scale ranging from 0 ("not true") to 2 ("certainly true"). The SDQ includes five subscales: Emotional symptoms, Conduct problems, Hyperactivity, Peer problems, and Prosocial behavior. This study specifically focuses on the two broader scales for Internalizing (combining Emotional symptoms and Peer problems subscales) and Externalizing (combining Conduct problems and Hyperactivity subscales) difficulties. The SDQ has been translated and adapted for use in the Greek population [46], with good psychometric properties.

Mothers evaluated their children’s internalizing and externalizing symptoms by means of the Child Behavior Checklist (CBCL) [47] at ages 6, 11, and 15. The CBCL, part of the Achenbach System of Empirically Based Assessment (ASEBA), evaluates both adaptive and maladaptive functioning in children aged 6 to 18 years. It comprises 113 items rated on a 3-point Likert scale from 0 ("not true") to 2 ("very often or often true"). The CBCL provides two main summaries: empirically based syndrome scales and DSM-oriented scales. This study specifically focuses on the broad-band scales for Internalizing (which include the Anxiety/Depression, Withdrawal/Depression, and Somatic Complaints syndrome scales) and Externalizing (which encompass the Rule-breaking Behavior and Aggressive Behavior syndrome scales) problems. The CBCL has been translated and adapted for Greek populations [48], demonstrating strong psychometric properties.

#### Children’s ADHD symptoms

Mothers assessed ADHD symptoms in their children using the Attention Deficit Hyperactivity Disorder Test (ADHDT) [49] when the children were 4 years old. The ADHDT is designed to evaluate ADHD-related symptoms in individuals aged 3 to 23 years, based on DSM-IV criteria. This instrument consists of 36 items rated on a Likert scale from 0 ("not a problem") to 2 ("severe problem"). The ADHDT provides three subscale scores—Hyperactivity, Inattention, and Impulsivity—as well as a total ADHD difficulties index, with scores ranging from 0 to 72, where higher scores indicate more severe symptoms. In the present analyses, the total ADHD difficulties index was utilized. The ADHDT has been translated and adapted for the Greek population by Maniadaki and Kakouros [50].

Mothers evaluated their children’s ADHD symptoms by means of the Conners’ Parent Rating Scale-Revised: Short Form (CPRS-R: S) [51] at ages 6, 11, and 15. The CPRS-R: S is a 27-item tool designed to assess ADHD symptoms. The instrument employs a 4-point Likert scale to rate the extent of difficulties experienced over the past month, with scores ranging from 0 ("not true at all") to 3 ("very much true"). It includes three subscales: Oppositional problems, Cognitive problems/Inattention, and Hyperactivity, along with a total ADHD symptoms index that ranges from 0 to 36, where higher scores indicate more severe symptoms. The total ADHD symptoms index was used in the present analyses. The CPRS-R: S has also been translated and adapted for the Greek population by the research team.

### Procedure

Children’s behavioral and emotional development was assessed longitudinally by trained psychologists using validated parental-report questionnaires to ensure accurate tracking. At each follow-up, mothers were contacted by phone to receive study updates and an invitation for their participation alongside their children. Participants completed self-administered questionnaires either in person or via a digital platform, as in the latest IntExt Trajectories project follow-up. Participants received detailed information about the study before providing written consent, and at the most recent follow-up, written consent was obtained separately from both mothers and their adolescent children. After participation, mothers received a personalized psychological report on their child’s development and had the option to schedule a counseling session with the Rhea team’s psychologists.

### Statistical analysis

Descriptive statistics were used to summarize the characteristics of the sample. Means with standard deviations (SD) and frequencies with percentages were calculated for continuous and categorical variables, respectively. To account for some missing data, total scores were prorated for all analyzed scales if less than 25% of the items were missing. We performed data harmonization of internalizing, externalizing and ADHD scores obtained from different instruments (ages 4, 6, 11, 15 years) with the following procedure. All raw prorated scales were categorized into four groups representing degrees of severity. We defined the degrees of severity as follows: no symptoms (scores below the 50th percentile), low symptoms (scores between the 51st and 75th percentile), moderate symptoms (scores between the 76th and 90th percentile), and severe symptoms (scores above the 90th percentile) [52–54].

To identify trajectories for various outcomes—internalizing, externalizing, and ADHD symptoms—Group-Based Trajectory Modeling (GBTM) was employed [55, 56]. Group-Based Trajectory Modeling (GBTM) is a statistical method that classifies individuals into distinct subgroups with similar developmental trajectories based on data-driven patterns, using maximum likelihood estimation for optimization. Although the observed variables may be continuous, GBTM is categorical in nature, as individuals are assigned to discrete trajectory groups. This approach allows for the identification of optimal trajectory groups without predefining them, providing a more objective alternative to traditional classification methods [55, 56]. The maximization is performed using a general quasi-Newton procedure [57].

We utilized the Stata Plugin traj [57, 58] to estimate the group-based trajectory models. The analysis calculated: a) the probability of group membership; b) the predicted trajectory for each group; and c) the posterior probabilities of group membership. The severity variables were modeled using a censored normal distribution [57], with censoring values set beyond the range of the data. We tested models with 2 to 4 possible trajectories for each single outcome trajectory model. We selected the optimal model based on the following criteria: a) the minimum Bayesian Information Criterion (BIC); b) a class membership greater than 5% for each group; c) an Average Posterior Probability (APP) greater than 0.7 for each group; d) Odds of Correct Classification (OCC) greater than 5 for each group [58, 59]; and e) entropy value greater than 0.80 that indicates that individuals are classified with confidence and there is adequate separation between the latent classes [60]. Given that different instruments (SDQ and ADHDT) were used at age 4 compared to later assessments (CBCL and CPRS-R), and while harmonization was attempted, it is likely that some of the identified trajectories may be influenced by differences in item content, scaling, and diagnostic scope across the instruments used. To assess the reliability of the scales in our sample, we computed Cronbach’s alpha values for each instrument, which were as follows: SDQ-internalizing 4 years (α = 0.56), SDQ-externalizing 4 years (α = 0.72), ADHDT 4 years (α = 0.95), CBCL-internalizing 6 years (α = 0.75), CBCL-externalizing 6 years (α = 0.87), CPRS-R 6 years (α = 0.83), CBCL-internalizing 11 years (α = 0.84), CBCL-externalizing 11 years (α = 0.88), CPRS-R 11 years (α = 0.85), CBCL-internalizing 15 years (α = 0.85), CBCL-externalizing 15 years (α = 0.89), and CPRS-R 15 years (α = 0.85). These values indicate that the scales used were sufficiently reliable for our analyses. To assess the impact of these differences across instruments, a sensitivity analysis was performed by excluding the age 4 data (where SDQ and ADHDT were used) and re-estimating the trajectory patterns using only the CBCL and CPRS-R data from later timepoints.

The univariate associations between early-life risk factors and group trajectories were examined using chi-square test for categorical variables (or the Fisher’s exact test in cases that chi-square assumptions were not satisfied) and analysis of variance (ANOVA) or Kruskal–Wallis for continuous variables. Variables found to be related to group trajectories with p < 0.05 in univariate analyses were then included in multivariate-adjusted models. The adjusted associations were examined by means of multinomial logistic regression models, weighted according to each individual’s posterior probability of belonging to each trajectory group. Separate models were built for each outcome (internalizing, externalizing, ADHD symptoms) and the largest group (*stable low*) was used as reference group. Based on prior literature, we examined potential non-linear associations between parental age and trajectories by including a quadratic term and maternal age was modeled using splines with a break point at 30 years for internalizing and 25 years for externalizing and ADHD symptoms. Given that approximately 84% of the participants had complete covariate data (leaving 16% with missing data), multiple imputation was used to handle the missing values. To increase sample size and reduce potential bias, missing data for the risk factors under study were imputed using multiple imputation with chained equations (MICE), generating twenty imputed datasets.

[61, 62]. This approach is appropriate for handling missing data in cases where the proportion of missing data is relatively low, as is the case in the present study. The imputation model included outcomes, covariates, and auxiliary variables [62]. Distributions in imputed datasets were similar to those observed (Table S1). Effect estimates from the imputed data sets were combined with the use of Rubin’s rules [61]. As a secondary analysis, we repeated the multinomial logistic regressions using alternative reference groups to explore whether early-life risk factors differentially predicted membership in specific trajectories. Effect estimates are presented in terms of relative risk ratios (RRRs) and 95% Confidence Intervals (CIs). Additionally, we examined potential effect modifications by sex, given the well-documented sex differences in emotional and behavioral problems. We introduced a multiplicative interaction term between each risk factor and child sex in the models. The statistical significance of the interaction was assessed through p values for the interaction term.

All hypothesis testing was conducted, assuming a 0.05 significance level and a two-sided alternative hypothesis. Stata version 16 (Stata Corp, College Station, TX) was used for all statistical analyses.

## Results

### Descriptives of the study population

The characteristics of the sample (N = 551) are presented in Table 1. The mean maternal and paternal age at childbirth was 30.1 years (± 4.7) and 34.0 years (± 5.6), respectively. Most mothers had medium (51.3%) or high (38.3%) educational level, while the corresponding percentages for fathers were 42.7% and 27.1%. The majority of women were employed during pregnancy (79.0%), married (89.8%), and lived in urban areas (79.0%) Among children, 52.1% were male and 47.9% were female, 66 (12.3%) were born prematurely, and nearly half (44.1%) were the firstborn in their families. By the age of 15 years, 33 children (6.0%) had been diagnosed with learning disabilities and 13 children (2.4%) had an ADHD diagnosis.

**Table 1:** Sociodemographic and perinatal characteristics of the sample (N = 551)

	N	% or Mean (SD)		N	% or Mean (SD)
Sociodemographic characteristics			Perinatal characteristics		
Maternal age at delivery (years)	546	30.1 (4.7)	Sex		
Missing (%)	5	0.9	Male	287	52.1
Paternal age at delivery (years)	528	34.0 (5.6)	Female	264	47.9
Missing (%)	23	4.2	Preterm birth (< 37 weeks)		
Maternal education			Yes	66	12.0
Low	56	10.2	No	471	85.5
Medium	277	50.3	Missing	14	2.5
High	207	37.6	Birth order		
Missing	11	2.0	First	210	38.1
Paternal education			Second	182	33.0
Low	158	28.7	Third or more	84	15.2
Medium	224	40.7	Missing	75	13.6
High	142	25.8	Mode of delivery		
Missing	27	4.9	Vaginal	279	50.6
Family origin			C-section	262	47.5
Both Greek	501	90.9	Missing	10	1.8
Both foreign/Mixed	32	5.8	Smoking status during pregnancy		
Missing	18	3.3	Nonsmokers	350	63.5
Maternal working status			Early pregnancy smokers	65	11.8
Employed	414	75.1	During pregnancy smokers	88	16.0
Not working/Unemployed	110	20.0	Missing	48	8.7
Missing	27	4.9	BMI pre-pregnancy (kg/m^2^)	526	24.9 (5.2)
Paternal working status			Missing (%)	25	4.5
Employed	526	95.5	Birth weight (kg)	534	3.2 (0.4)
Unemployed	3	0.5	Missing (%)	17	3.1
Missing	22	4.0	Birth length (cm)	526	50.5 (2.3)
Maternal marital status			Missing (%)	25	4.5
Married	475	86.2	Birth head circumference (cm)	519	34.2 (1.4)
Other	54	9.8	Missing (%)	32	5.8
Missing	22	4.0	Gestational age (weeks)	537	38.2 (1.6)
Parity			Missing (%)	14	2.5
Nulliparous	256	46.5	Intensive Care Unit		
Multiparous	291	52.8	Yes	91	16.5
Missing	4	0.7	No	407	73.9
Household income (€, median, IQR)	461	1040.7 (511.9)	Missing	53	9.6
1st tertile	153	27.8	Breastfeeding duration (months)	520	4.2 (4.1)
2nd tertile	154	27.9	Missing (%)	31	5.6
3rd tertile	154	27.9	Child characteristics		
Missing	90	16.3	Exact age at assessment		
Area of living			4 years	531	4.2 (0.2)
Urban	377	68.4	6 years	462	6.6 (0.3)
Rural	100	18.1	11 years	328	11.0 (0.3)
Missing	74	13.4	15 years	475	15.0 (0.4)
			Diagnosis by age 15 years		
			None	505	91.7
			Learning disabilities	33	6.0
			ADHD	13	2.4

BMI: Body Mass Index

The non-response analysis showed some differences between participants (n = 551) and non-participants (n = 446) (Table S2). Mothers included in the analysis were more likely to have high educational level, which was observed among fathers as well. Additionally, participating mothers were more likely to be employed during pregnancy, to report higher household income, and higher pre-pregnancy BMI compared to non-participants. Participating children were less likely to be delivered through cesarian section and were found to have slightly higher birth measurements and longer breastfeeding durations.

### Descriptives of the study outcome variables for the total sample and by sex

The distribution of internalizing, externalizing, and ADHD symptoms is shown in Table 2. At age 4, males had higher scores for internalizing problems than females, but this difference was not significant at ages 6 and 11. By age 15, females reported significantly more internalizing symptoms. For externalizing difficulties, males had higher scores at all assessments except at age 15. Males also consistently exhibited higher levels of ADHD symptoms than females at all timepoints.

**Table 2:** Distribution of the study outcomes (raw scores) for the total sample (N = 551) and by gender

	Overall				Males		Females		p-value
	N	Mean (SD)	Min	Max	N	Mean (SD)	N	Mean (SD)	
Internalizing symptoms									
SDQ 4 years	514	3.2 (2.4)	0	17	262	3.4 (2.6)	252	2.9 (2.2)	0.026
CBCL 6 years	447	5.9 (4.3)	0	30	237	6.2 (4.5)	210	5.5 (4.0)	0.064
CBCL 11 years	326	7.2 (5.7)	0	34	180	7.6 (6.0)	146	6.7 (5.2)	0.165
CBCL 15 years	473	6.9 (5.8)	0	29	239	6.1 (5.3)	234	7.7 (6.1)	0.003
Externalizing symptoms									
SDQ 4 years	513	5.3 (3.2)	0	18	262	5.7 (3.4)	251	4.8 (2.8)	0.001
CBCL 6 years	449	8.4 (6.3)	0	38	238	9.7 (6.6)	211	6.9 (5.6)	< 0.001
CBCL 11 years	326	7.2 (6.3)	0	36	180	8.1 (7.2)	146	6.0 (4.8)	0.003
CBCL 15 years	472	6.5 (6.2)	0	42	239	6.6 (6.1)	233	6.3 (6.3)	0.646
ADHD symptoms									
ADHDT 4 years	512	14.8 (12.1)	0	62	261	16.8 (13.3)	251	12.8 (10.2)	< 0.001
CPRS 6 years	444	8.8 (5.5)	0	34	237	9.6 (5.7)	207	7.8 (5.1)	< 0.001
CPRS 11 years	328	8.4 (5.8)	0	34	181	9.2 (6.3)	147	7.4 (4.8)	0.004
CPRS 15 years	474	7.9 (5.8)	0	32	239	8.8 (6.0)	235	6.9 (5.4)	< 0.001

ADHDT: Attention Deficit Hyperactivity Disorder Test; CBCL: Child Behavior Checklist; CPRS: Conners’ Parent Rating Scale; SDQ: Strengths and Difficulties Questionnaire Bold font indicates p < 0.05

### Identification of trajectory groups

Different models were tested using GBTM, with the number of trajectories ranging from two to four. For each outcome, the four-trajectory model was considered optimal. Table 3 presents the data for the selected models. For internalizing symptoms, two trajectories were shaped to be zero-order and two linear, while for externalizing symptoms one trajectory was shaped to be zero-order and three were shaped to be linear. Regarding ADHD symptoms, two trajectories were zero-order and two were quadratic. The models with the lowest BIC values were selected and BIC values for the internalizing, externalizing and ADHD symptoms models were -2241.18, -2183.53, and -2231.22, respectively. Entropy ranged from 0.817 to 0.846. Average posterior probabilities were above the criterion of 0.70, ranging from 0.81 to 0.95. Odds of correct classification were greater than 8.4. Individual trajectories based on GBTM analysis are presented for each outcome in (Figure S2).

**Table 3:** Data for selected trajectories

				Probability				Average posterior probability				Odds of correct classification			
Outcome	Shape^a^	BIC	Entropy	G1	G2	G3	G4	G1	G2	G3	G4	G1	G2	G3	G4
Internalizing symptoms	0 1 1 0	-2241.18	0.817	63.4	16.1	11.7	8.8	0.94	0.85	0.81	0.88	8.4	28.9	36.3	79.0
Externalizing symptoms	1 1 1 0	-2183.53	0.846	17.2	59.7	13.4	9.7	0.86	0.95	0.87	0.91	30.6	12.9	43.6	88.5
ADHD symptoms	2 0 2 0	-2231.22	0.823	15.2	59.0	16.3	9.4	0.85	0.94	0.84	0.88	34.5	9.9	28.1	65.5

BIC: Bayesian Information Criterion; G: Group

^**a**^ Shape: 0 = zero-order, 1 = linear, 2 = quadratic

**Fig. 2:**
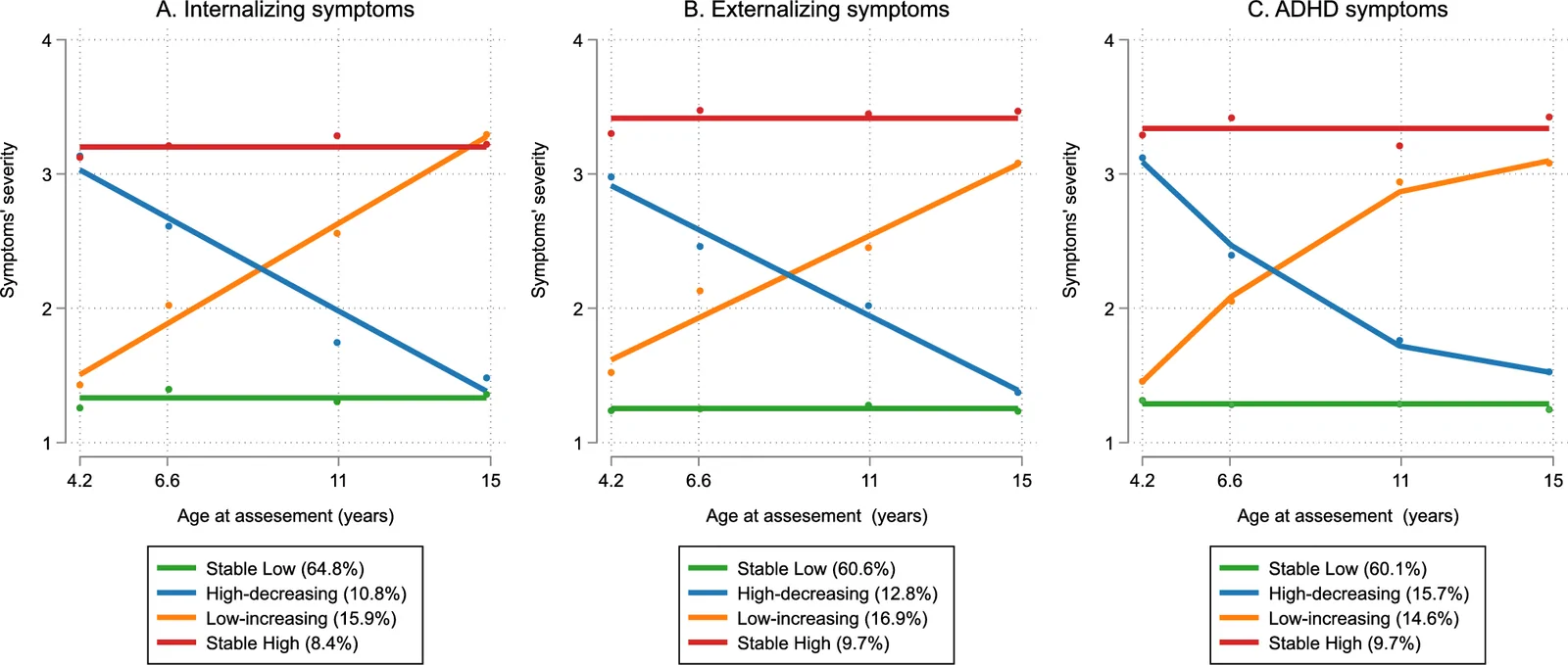
Trajectory groups of (**a**) internalizing, (**b**) externalizing, and (**c**) ADHD symptoms from age 4 to 15 years

The distinct trajectory groups identified for internalizing, externalizing, and ADHD symptoms are illustrated in Fig. 2. The majority of children were assigned to the *stable low* group (internalizing: Ν = 354, 64.8%; externalizing: Ν = 330, 60.6%; ADHD: Ν = 330, 60.1%), which exhibited consistently low severity of symptoms from early childhood up to adolescence. Children in the *high-decreasing* trajectory (internalizing: Ν = 59, 10.8%; externalizing: Ν = 70, 12.8%; ADHD: Ν = 86, 15.7%) presented high symptoms during early childhood, which gradually declined by the age of 15 years. The reverse course was observed for children included in the *low-increasing* group (internalizing: Ν = 87, 15.9%; externalizing: N = 92, 16.9%; ADHD: N = 80, 14.6%), as symptom severity was low at 4 years, but increased until adolescence. Lastly, the *stable high* group (internalizing: N = 46, 8.4%; externalizing: N = 53, 9.7%; ADHD: N = 53, 9.7%) represented a minority of the sample and consisted of children who showed persistently high symptom levels from 4 to 15 years of age.

The sensitivity analysis, excluding the age 4 data, revealed trajectory patterns that were largely consistent with those derived from the full sample (Figure S2). The four-trajectory models were again selected for each outcome based on statistical criteria. For externalizing and ADHD symptoms, the trajectory shapes and group distributions closely aligned with the original models. However, for internalizing symptoms, minor variations were observed, including steeper increases or decreases in the fluctuating groups. Additionally, a higher proportion of children were assigned to the stable high group, which showed a slight increase in symptom severity from ages 6 to 11. These findings suggest that instrument differences at age 4 had minimal impact on the overall trajectory patterns (Table S3).

### Early-life sociodemographic and perinatal risk factors and developmental trajectories, univariate analyses

In univariate analyses (Tables S4-S6), maternal and paternal age, child sex and birth length were related to the group trajectories for internalizing symptoms. Maternal age, maternal and paternal education, maternal employment status during pregnancy, family income, smoking status during pregnancy, child sex and breastfeeding duration were found to have a significant association with developmental trajectories for externalizing difficulties. Lastly, the factors that were associated with ADHD symptom trajectories were maternal age at childbirth, paternal educational level, maternal employment status, household income, child sex and breastfeeding duration.

### Early-life sociodemographic and perinatal risk factors and developmental trajectories, multivariable analyses

Table 4 presents the results of multivariable analysis between early-life sociodemographic and perinatal risk factors and developmental trajectories of internalizing, externalizing and ADHD symptoms. Parental age and child’s sex were found to be significant predictors of internalizing symptom trajectories. Up to age 30, higher maternal age at delivery was associated with lower probability of membership in the *high-decreasing* trajectory (RRR [95% CI]: 0.86 [0.80, 0.93]), while above 30 no significant associations with trajectory groups were observed. Higher paternal age at delivery was associated with lower probability of membership in the *low-increasing* trajectory (RRR [95% CI]: 0.93 [0.88, 0.98]). Female offsprings were less likely to be assigned in the *high-decreasing* group of internalizing symptoms compared to males (RRR [95% CI]: 0.46 [0.29, 0.73]). However, they were also more likely to follow the trajectory of *low-increasing* internalizing symptoms (RRR [95% CI]: 1.66 [1.09, 2.52]).

**Table 4:** Adjusted associations of sociodemographic and perinatal factors with trajectory groups of internalizing, externalizing, and ADHD symptoms, multinomial logistic regression models

	Internalizing symptoms (N=546)			Externalizing symptoms (N=545)			ADHD symptoms (N=549)		
	High-decreasing	Low-increasing	Stable High	High-decreasing	Low-increasing	Stable High	High-decreasing	Low-increasing	Stable High
	RRR (95% CI)	RRR (95% CI)	RRR (95% CI)	RRR (95% CI)	RRR (95% CI)	RRR (95% CI)	RRR (95% CI)	RRR (95% CI)	RRR (95% CI)
Maternal age (years)									
Spline 1*	0.86 (0.80, 0.93)	1.02 (0.93, 1.11)	0.92 (0.83, 1.01)	0.77 (0.65, 0.92)	1.12 (0.87, 1.44)	0.83 (0.67, 1.03)	0.80 (0.67, 0.96)	0.89 (0.72, 1.12)	0.71 (0.57, 0.89)
Spline 2**	1.05 (0.95, 1.16)	1.07 (0.96, 1.19)	0.99 (0.87, 1.12)	1.01 (0.94, 1.07)	0.94 (0.88, 1.00)	0.99 (0.91, 1.07)	0.94 (0.89, 1.01)	0.97 (0.91, 1.03)	1.05 (0.98, 1.13)
Paternal age (years)	0.99 (0.94, 1.04)	0.93 (0.88, 0.98)	0.97 (0.90, 1.04)	-	-	-	-	-	-
Maternal education									
Medium (ref.: Low)	-	-	-	0.79 (0.39, 1.63)	0.80 (0.40, 1.60)	1.66 (0.56, 4.89)	0.75 (0.38, 1.47)	0.66 (0.31, 1.39)	1.20 (0.40, 3.62)
High (ref.: Low)	-	-	-	0.55 (0.23, 1.30)	0.75 (0.31, 1.82)	2.37 (0.65, 8.60)	0.89 (0.39, 2.01)	0.75 (0.31, 1.82)	1.75 (0.47, 6.48)
Paternal education	-	-	-						
Medium (ref.: Low)	-	-	-	0.86 (0.50, 1.50)	0.77 (0.46, 1.30)	**0.36 (0.19, 0.70)**	0.71 (0.44, 1.14)	1.58 (0.90, 2.76)	0.71 (0.36, 1.41)
High (ref.: Low)	-	-	-	0.68 (0.31, 1.49)	0.89 (0.44, 1.79)	0.45 (0.17, 1.18)	0.69 (0.34, 1.41)	1.55 (0.76, 3.16)	0.62 (0.22, 1.71)
Maternal working status (pregnancy)									
Working (ref.: Unemployed)	-	-	-	0.97 (0.52, 1.80)	1.23 (0.67, 2.26)	0.53 (0.23, 1.21)	0.73 (0.42, 1.29)	1.18 (0.63, 2.21)	1.67 (0.65, 4.25)
Household income (tertiles)									
2^nd^ Tertile (ref.: 1^st^)	-	-	-	1.44 (0.74, 2.81)	1.20 (0.64, 2.27)	1.53 (0.63, 3.71)	1.07 (0.59, 1.93)	1.26 (0.63, 2.49)	0.91 (0.37, 2.21)
3^rd^ Tertile (ref.: 1^st^)	-	-	-	0.71 (0.30, 1.69)	0.69 (0.29, 1.67)	0.64 (0.20, 2.06)	0.67 (0.28, 1.65)	0.59 (0.25, 1.38)	0.42 (0.13, 1.32)
Smoking status during pregnancy									
Early pregnancy smokers (ref.: Nonsmokers)	-	-	-	1.11 (0.55, 2.25)	1.08 (0.58, 2.00)	0.97 (0.35, 2.65)	-	-	-
During pregnancy smokers (ref.: Nonsmokers)	-	-	-	1.77 (0.96, 3.26)	1.09 (0.58, 2.05)	**2.28 (1.05, 4.93)**	-	-	-
Sex									
Female (ref.: Male)	**0.46 (0.29, 0.73)**	**1.66 (1.09, 2.52)**	0.76 (0.42, 1.36)	**0.42 (0.26, 0.68)**	0.74 (0.49, 1.12)	**0.35 (0.19, 0.64)**	**0.52 (0.34, 0.79)**	**0.59 (0.39, 0.91)**	**0.33 (0.19, 0.60)**
Breastfeeding duration (months)	-	-	-	0.98 (0.91, 1.05)	1.00 (0.95, 1.06)	0.99 (0.92, 1.07)	**0.92 (0.86, 0.98)**	0.98 (0.93, 1.04)	0.96 (0.89, 1.04)
Birth length (per 1cm increase)	0.99 (0.90, 1.08)	0.98 (0.89, 1.08)	1.11 (0.98, 1.26)	-	-	-	-	-	-

Reference: Stable Low trajectory. Effect estimates are based on 20 imputed datasets using Rubin’s rules Bold font indicates p < 0.05.

*Spline 1: maternal age ≤ 30 for internalizing symptoms and ≤ 25 for externalizing and ADHD symptoms.

**Spline 2: maternal age > 30 for internalizing symptoms and > 25 for externalizing and ADHD symptoms.

Regarding externalizing symptoms, increasing maternal age at delivery was associated with lower probability of membership in the *high-decreasing* trajectory only up to age 25 (RRR [95% CI]: 0.77 [0.65, 0.92]). Above age 25, an increasing maternal age was associated with lower probability for *low-increasing* behavioral difficulties (RRR [95% CI]: 0.94 [0.88, 1.00]). Children of fathers with medium educational level were at lower risk of having persistently high behavioral symptoms (RRR [95% CI]: 0.36 [0.19, 0.70]). Maternal smoking during pregnancy was associated with higher probability of exhibiting *stable high* symptoms (RRR [95% CI]: 2.28 [1.05, 4.93]). Finally, females were less likely to be assigned in the *high-decreasing* and the *stable high* trajectory groups for externalizing problems compared to males (RRR [95%CI]: 0.42 [0.26, 0.68], 0.35 [0.19, 0.64], respectively).

Concerning ADHD symptoms, higher maternal age at delivery up to age 25 was associated with lower probability of membership in the *high-decreasing* and the *stable high* group (RRR [95% CI]: 0.80 [0.67, 0.96], 0.71 [0.57, 0.89], respectively), with no significant associations observed for maternal age above 25 years. Females were less likely to be assigned to any of the ADHD trajectory groups compared to the *stable low* group (RRR [95% CI]: 0.52 [0.34, 0.79] for *high-decreasing*, 0.59 [0.39, 0.91] for *low-increasing*, 0.33 [0.19, 0.60] for *stable high*). Children who were breastfed for a longer duration were less likely to be assigned in the *high-decreasing* group (RRR [95% CI]: 0.92 [0.86, 0.98]).

When analyses were repeated using alternative reference groups, additional insights emerged regarding the ability of early-life risk factors to distinguish between trajectories (Table S7). For internalizing symptoms, increasing maternal age up to age 30 was associated with higher probability of following the *low-increasing* trajectory rather than the *high-decreasing* group. The association between female sex and increased likelihood of *low-increasing* emotional symptoms remained robust across all comparisons. For externalizing symptoms, increasing maternal age until age 25 was linked to a higher probability of *low-increasing* symptoms compared to both the *high-decreasing* and *stable high* trajectories. High maternal education was associated with a lower likelihood of *high-decreasing* rather than *stable high* symptoms, while medium paternal education was linked to a higher probability of fluctuating symptoms compared to persistently high problems. Girls were consistently more likely to exhibit *low-increasing* symptoms. Increasing maternal age beyond 25 years was associated with a higher probability for persistently high ADHD symptoms compared to both the *low-increasing* and the *high-decreasing* trajectories. Medium paternal education was consistently linked to a higher likelihood of *low-increasing* symptoms.

No significant interaction effects of sex were observed in the models, indicating that the relationship between risk factors and developmental trajectories was consistent across both males and females (Table S8).

## Discussion

The present study examined the developmental trajectories of internalizing, externalizing, and ADHD symptoms in Greek children aged 4 to 15, identifying four distinct patterns: *stable low, high-decreasing, low-increasing* and *stable high*. Furthermore, this study highlights several early-life sociodemographic and perinatal factors, such as child’s sex, maternal and paternal age, paternal education, maternal smoking during pregnancy, and duration of breastfeeding that are significantly linked to the development of internalizing, externalizing, and ADHD symptoms.

Our study identified four distinct symptom trajectories for internalizing, externalizing, and ADHD symptoms, indicating that symptom patterns evolve over time. As expected in a population study, the majority of children (64.8% for internalizing, 60.6% for externalizing, and 60.1% for ADHD) followed the *stable low* trajectory, showing consistently low symptoms from early childhood to adolescence, which is common in population-based studies [8, 10, 16]. A smaller proportion exhibited the *high-decreasing* trajectory (10.8% for internalizing, 12.8% for externalizing, and 15.7% for ADHD), where symptoms were initially high but gradually declined, while others followed the *low-increasing* trajectory (15.9% for internalizing, 16.9% for externalizing, and 14.6% for ADHD), where symptoms gradually increased from a low baseline. Similar trajectory patterns have been observed in longitudinal studies from the UK, the US, and other European cohorts, although their prevalence and shape may vary depending on sociocultural context and reporting practices [13, 14, 17]. However, some differences across studies have been reported, such as Gutman and Codiroli McMaster [11] not identifying a decreasing trajectory for emotional symptoms in girls, and Papachristou and Flouri [10] observing an increasing trajectory for initially high behavioral problems. The smallest subgroup in our study, the *stable high* group (8.4% for internalizing, 9.7% for externalizing, and 9.7% for ADHD), showed persistently high levels of symptoms across development, which aligns with findings from prior research [12, 14, 15, 17]. Taken together, these results highlight the diverse trajectories in symptom development, with most children demonstrating stable or improving patterns, while a minority showed escalating or persistently high symptoms, consistent with earlier research on the stability and change of mental health symptoms in childhood and adolescence [7, 8, 16, 17]. In the Greek context, strong family ties and extended kin networks may help buffer symptom severity for some children, potentially contributing to the relatively high proportion of stable low trajectories, while simultaneously increasing pressure and expectations for others [63].

Mental health conditions like ADHD and anxiety, often emerge in early childhood, a critical period marked by rapid brain development and the onset of cognitive, emotional, and behavioral regulation. Between ages 3 and 6, disruptions in these developmental processes can lead to the early onset of both internalizing and externalizing symptoms. These conditions may evolve and become more complex as children progress into later childhood and adolescence. Research, such as the meta-analysis by Solmi et al. [64], highlights that many mental health disorders begin early in childhood, often around ages 5 to 6. For example, ADHD and most anxiety disorders are known to peak in onset around 5.5 years of age, which coincides with the early years covered in our study. This early onset aligns with critical developmental transitions between childhood and adolescence; periods marked by significant brain changes and shifts in social and environmental factors. As such, understanding when and how disorders typically emerge can provide important insights into the trajectory of symptoms over time and help inform early detection and intervention strategies. Moreover, cross-national research suggests that while the timing of onset is broadly similar across countries, cultural factors, such as parental expectations, educational demands, and stigma surrounding mental health, may influence how symptoms are expressed, perceived, and reported [65, 66].

Our study highlights significant gender differences in symptom trajectories, with distinct patterns emerging for internalizing and externalizing behaviors. Girls were more likely to exhibit *low-increasing* internalizing symptoms, while boys had higher probability to follow the *high-decreasing* trajectory of internalizing symptoms. Our findings suggest that females experience a later onset of emotional difficulties and a higher prevalence of depressive symptoms after puberty, supporting existing literature [54, 67]. This is consistent with previous research indicating that girls are more susceptible to adolescence-onset internalizing disorders and tend to show an increasing trajectory of depressive symptoms compared to boys [13, 68]. In Greece, traditional gender roles and cultural expectations emphasizing emotional restraint and caregiving among females may further contribute to the gradual escalation of internalizing symptoms during adolescence [69]. By contrast, males tend to exhibit higher emotional symptoms in early childhood that gradually diminish during adolescence, aligning with research suggesting that emotional symptoms are more common in males before puberty [67, 70]. Beyond internalizing symptoms, boys were more likely to exhibit *high-decreasing* and *stable high* externalizing symptoms, as well as follow adverse ADHD-related trajectories (*high-decreasing, low-increasing, stable high*). This aligns with research highlighting their greater vulnerability to behavioral disorders such as aggression, rule-breaking, hyperactivity, and inattention [23, 67], as well as persistent ADHD symptoms [42]. The strong emphasis placed on academic performance within the Greek educational system may disproportionately affect boys with attentional or behavioral difficulties, potentially reinforcing persistent or escalating symptom trajectories [66].

Our findings suggest that sociodemographic factors, such as parental age, significantly influence child development. Specifically, higher maternal age at delivery was associated with a lower risk of adverse trajectories in internalizing, externalizing and ADHD symptoms, aligning with previous research linking younger maternal age to increased psychopathology risk in offspring [23, 24, 71, 72]. Unhealthy behaviors during pregnancy, like smoking, and less nurturing parenting practices in younger mothers may create unstable child-rearing environments [73], though more research is needed to clarify the link between maternal age and child behavioral symptoms. Importantly, the protective effect of increased maternal age against internalizing and ADHD symptoms was observed only up to 30 and 25 years of age, respectively, with no significant associations at older maternal ages, suggesting a nonlinear relationship between maternal age and child outcomes. This pattern aligns with findings from a large population-based study, which reported that both younger and older parental ages were linked to increased risk of psychiatric disorders [74]. In Greece, younger parental age often coincides with greater socioeconomic instability and limited access to supportive resources, which may further amplify developmental risks [75]. Our results also indicate that higher paternal age is linked to a reduced risk of *low-increasing* emotional symptoms. While existing evidence suggests that younger paternal age is a risk factor for emotional disorders with onset in childhood and adolescence [74], other studies have not found a significant association between paternal age and internalizing problems [72, 76], highlighting the need for further research, particularly on symptom progression over time.

Our findings suggest that children of fathers with a medium level of education are less likely to exhibit persistently high behavioral symptoms. While much of the existing research focuses on maternal education, studies also highlight the positive influence of paternal education on reducing externalizing and ADHD-related issues in children and adolescents [25, 77]. Within the Greek cultural context, paternal education may be particularly relevant given traditional family structures in which fathers often play a central role in economic stability and decision-making [63]. Fathers with higher education levels may also engage in more positive parenting practices and experience less marital conflict, both of which contribute to better mental health outcomes for their children [78]. However, further research is needed to fully understand the underlying mechanisms that drive these patterns.

Maternal smoking during pregnancy was identified as a significant predictor of externalizing symptoms, particularly linked to the *stable high* trajectory. Previous studies have consistently shown maternal smoking as a risk factor for externalizing behaviors [34, 36, 79]. However, contrary to our findings, Sutin et al. [80] found that maternal smoking was associated with an increase in behavioral symptoms from early childhood to adolescence, but only when symptoms were reported by teachers rather than parents, as in our study. Prenatal exposure to smoke has been shown to alter brain structure and function [81] and is also linked to reduced maternal emotional connection to the fetus [82]. Additionally, prior studies have shown that personality traits, such as neuroticism and extraversion, are associated with smoking behavior [83], and these traits are also linked to children’s emotional and behavioral development [84]. Evidence suggests that the relationship between maternal smoking during pregnancy and offspring behavioral outcomes may be largely attributable to genetic factors, which influence both maternal and child behavior [85]. Consequently, the observed association between maternal smoking and externalizing symptoms may be influenced by unmeasured confounding factors, including shared genetic and environmental influences. Future research utilizing genetically informed or quasi-experimental designs is needed to more clearly unravel the complex pathways through which maternal smoking affects child outcomes.

Finally, our results indicate that longer breastfeeding duration serves as a protective factor against the manifestation of *high-decreasing* ADHD symptoms. Previous research has shown that breastfeeding is associated with fewer attention and hyperactivity symptoms in children [86] and lower risk of ADHD [87]. Large cohort studies have also highlighted its long-term benefits for behavioral development [37, 38]. In addition to providing essential nutrients and bioactive factors critical for offspring’s growth and immune protection [88], breastfeeding enhances mother–child bonding and promotes infant’s secure attachment [89, 90]. However, it should be noted that the duration a mother breastfeeds her child depends on several factors, such as maternal traits and sociodemographic factors [91, 92], which may also have direct effects on child outcomes. Thus, the observed association between breastfeeding and ADHD symptoms should be interpreted with caution, as it may be confounded by these underlying influences. Our findings suggest that children breastfed for a shorter period are at greater risk of presenting ADHD symptoms in early childhood, underscoring the importance of early interventions.

Comparisons with varying reference groups generally confirmed the findings of the main analysis, while providing additional insight for targeted prevention and intervention strategies. The consistent association between female sex and low-increasing emotional symptoms highlights the need of early monitoring and support for girls, even though symptom severity may increase gradually over time. Similarly, maternal age at childbirth emerged as a significant predictor across internalizing, externalizing, and ADHD symptom trajectories. Children of both younger and older mothers may follow distinct developmental pathways, emphasizing the importance of tailored support services to promote better developmental outcomes across diverse family contexts.

### Strengths and limitations

A strength of this study is its use of person-oriented approaches, such as GBTM, to identify distinct subgroups within the population and examine their unique developmental patterns. This method provides a more nuanced understanding of individual variability and symptom progression over time, offering insights into the heterogeneity of symptom development that traditional variable-oriented approaches may miss. Additionally, the study utilized data from the well-established Rhea mother–child cohort, with internalizing, externalizing, and ADHD symptoms assessed through valid and reliable psychometric tools. Finally, analyzing ADHD separately from broader externalizing symptoms allowed for a clearer understanding of its unique developmental trajectory. This approach highlights ADHD’s early onset and its distinct impact on symptom progression, ensuring its specific influence is not obscured by other externalizing behaviors, and emphasizing the importance of treating ADHD as a separate construct in developmental studies.

However, there are several limitations to consider. First, while GBTM provides unique insights by identifying distinct developmental subgroups, it may have limitations in clinical applicability compared to variable-centered models like growth curve modeling, which focus on average trends and individual variation across the entire sample. Using GBTM allows for a more nuanced understanding of specific trajectories, but the results may not fully capture the complexity of individual change, as the identified groups are statistical constructs. Second, different instruments were used to assess outcomes at various timepoints (SDQ and ADHDT at age 4, CBCL and CPRS-R:S at later timepoints), which may have influenced symptom trajectories due to differences in item content, scaling, and diagnostic breadth. While data harmonization was applied and a sensitivity analysis excluding age 4 data showed consistent results, these instrument differences may still have impacted the classification of symptom trajectories. Third, potential selection bias may arise from the inclusion criterion requiring at least two assessments per participant, which could exclude children with missing data from one of the timepoints, potentially affecting the generalizability of the findings. Although a non-response analysis showed only minor differences in socio-economic status, birth measurements, and breastfeeding duration, future research could explore the impact of varying inclusion criteria to determine whether more inclusive approaches could reduce bias and increase statistical power. Fourth, raw scores were used for symptom scales instead of T-scores or standardized scores, which may be influenced by sex-related differences in scale norms and could affect the interpretation of sex differences. Fifth, no formal corrections for multiple testing, such as Bonferroni or False Discovery Rate (FDR), were applied in this study due to its exploratory nature. However, we acknowledge that multiple comparisons increase the risk of Type I error, and caution is needed when interpreting results, particularly those with borderline significance. Consistent patterns observed across related outcomes offer stronger confidence in these associations. Future research should consider using more conservative approaches or hypothesis-driven analyses to validate these findings. Lastly, symptom assessments were based on maternal reports rather than clinical diagnoses, and discrepancies between informants in symptom reporting are well-documented [93], which could have affected data accuracy. Excluding children with autism spectrum disorder (n = 11) may limit the generalizability of the findings, as it could underrepresent trajectory patterns that overlap with or differ from those seen in ADHD and other disorders. However, since our measures were not designed to capture autism spectrum disorder-specific outcomes, including these cases could have introduced heterogeneity, potentially obscuring the patterns under investigation. Future research including children with autism spectrum disorder would be valuable to explore potential overlaps or differences in developmental trajectories.

## Conclusion

In conclusion, this study offers valuable insights into the developmental trajectories of internalizing, externalizing, and ADHD symptoms in children, highlighting the importance of considering sociodemographic and perinatal factors in understanding these patterns. A multifaceted approach to child behavioral development can enhance prevention, diagnosis, and treatment efforts, leading to better outcomes as children navigate growth challenges. Future research should explore the mechanisms underlying distinct symptom trajectories, focusing on the interplay of biological, environmental, and sociodemographic factors, and investigate effective, gender-specific interventions targeting early risk factors for improved mental health outcomes. Additionally, future studies should examine whether children belong to multiple elevated-risk groups (e.g., high internalizing and high ADHD) to better understand the overlap across symptom trajectories and identify those who may experience broad-spectrum difficulties. Future research should explore how cultural and societal factors interact with socio-demographic and perinatal risk factors to better understand their influence on child and adolescent mental health trajectories.

## Supplementary Information

Below is the link to the electronic supplementary material.Supplementary file1 (DOCX 328 KB)

## Data Availability

The data that support the findings of this study may be provided by the corresponding author upon reasonable request.
