# Supplementary material for: Early-life factors associated with developmental trajectories of internalizing and externalizing symptoms from early childhood through adolescence: Rhea mother—child cohort in Crete, Greece
Source: Soc Psychiatry Psychiatr Epidemiol. 2026 May 11;61(8):1523–40. doi: 10.1007/s00127-026-03103-6 (PMC13522092; doi:10.1007/s00127-026-03103-6)
Supplement: Supplementary file 1 [file 127_2026_3103_MOESM1_ESM.docx]

**Early-life factors associated with developmental trajectories of internalizing, externalizing and ADHD symptoms from early childhood through adolescence: Rhea mother - child cohort in Crete, Greece**

Social Psychiatry and Psychiatric Epidemiology

Katerina Koutra^1^, Chrysi Mouatsou^1^, Katerina Margetaki^2^, Theano Roumeliotaki^2^, Sofia Psoma^1^, Mariza Kampouri^3^, Lida Chatzi^4^

^1^Department of Psychology, School of Social Sciences, University of Crete, Rethymno, Crete, Greece

^2^Clinic of Ρreventive and Social Medicine, Department of Social Medicine, Faculty of Medicine, University of Crete, Heraklion, Crete, Greece

^3^Institute of Environmental Medicine, Karolinska Institutet, Stockholm, Sweden

^4^Department of Preventive Medicine, Keck School of Medicine, University of Southern California, Los Angeles, USA

*Corresponding Author

Katerina Koutra, PhD, Associate Professor in Clinical Psychology

Department of Psychology, School of Social Sciences, University of Crete, Greece, Gallos Campus 74100, Rethymno, Crete, Greece. Telephone: ++30 2810324999/++30 6977357108, e-mail: [koutra.k@gmail.com](mailto:koutra.k@gmail.com), [kkoutra@uoc.gr](mailto:kkoutra@uoc.gr)

**Table S1** Sociodemographic and perinatal characteristics of the sample in the observed data compared to the imputed data (N=551, 20 imputed datasets)

|  | **Observed Data** | | **Imputed Data** |
| --- | --- | --- | --- |
|  | **N** | **% or**  **Mean (SD)** | **% or**  **Mean (SE)** |
| **Sociodemographic characteristics** |  |  |  |
| **Maternal age at delivery** (years) | 546 | 30.1 (4.7) | 30.0 (0.2) |
| **Paternal age at delivery** (years) | 528 | 34.0 (5.6) | 34.0 (0.2) |
| **Maternal education** |  |  |  |
| Low | 56 | 10.4 | 10.4 |
| Medium | 277 | 51.3 | 51.4 |
| High | 207 | 38.3 | 38.2 |
| **Paternal education** |  |  |  |
| Low | 158 | 30.2 | 30.3 |
| Medium | 224 | 42.7 | 42.7 |
| High | 142 | 27.1 | 27.0 |
| **Family origin** |  |  |  |
| Both Greek | 501 | 94.0 | 95.4 |
| Both foreign/Mixed | 32 | 6.0 | 4.6 |
| **Maternal working status** |  |  |  |
| Employed | 414 | 79.0 | 79.0 |
| Not working/Unemployed | 110 | 21.0 | 21.0 |
| **Maternal marital status** |  |  |  |
| Married | 475 | 89.8 | 89.8 |
| Other | 54 | 10.2 | 10.2 |
| **Parity** |  |  |  |
| Nulliparous | 256 | 46.8 | 46.9 |
| Multiparous | 291 | 53.2 | 53.1 |
| **Household income** (€, median, IQR) | 461 | 1040.7 (511.9) | 1038.4 (505.0) |
| 1^st^ tertile | 153 | 33.2 | 33.2 |
| 2^nd^ tertile | 154 | 33.4 | 34.0 |
| 3^rd^ tertile | 154 | 33.4 | 32.7 |
| **Area of living** |  |  |  |
| Urban | 377 | 79.0 | 79.1 |
| Rural | 100 | 21.0 | 20.9 |
| **Perinatal characteristics** |  |  |  |
| **Preterm birth** (<37 weeks) |  |  |  |
| Yes | 66 | 12.3 | 12.5 |
| No | 471 | 87.7 | 87.5 |
| **Mode of delivery** |  |  |  |
| Vaginal | 279 | 51.6 | 51.6 |
| C-section | 262 | 48.4 | 48.4 |
| **Smoking status during pregnancy** |  |  |  |
| Nonsmokers | 350 | 69.6 | 69.2 |
| Early pregnancy smokers | 65 | 12.9 | 13.0 |
| During pregnancy smokers | 88 | 17.5 | 17.8 |
| **Birth weight** (kg) | 534 | 3.2 (0.4) | 3.2 (0.02) |
| **Birth length** (cm) | 526 | 50.5 (2.3) | 50.5 (0.1) |
| **Gestational** **age** (weeks) | 537 | 38.2 (1.6) | 38.2 (0.1) |
| **Breastfeeding duration** (months) | 520 | 4.2 (4.1) | 4.2 (0.2) |

*Abbreviations*: BMI: Body Mass Index.

**Table S2** Sociodemographic and perinatal characteristics of the sample included in GBTM analysis (N=551) compared to those excluded from analyses (N=446)

|  | **Participants** | | **Non-participants** | |  |
| --- | --- | --- | --- | --- | --- |
|  | **N** | **% or Mean (SD)** | **N** | **% or Mean (SD)** | **p-value** |
| **Sociodemographic characteristics** |  |  |  |  |  |
| **Maternal age at delivery** (years) | 546 | 30.1 (4.7) | 416 | 29.7 (5.2) | 0.237 |
| **Paternal age at delivery** (years) | 528 | 34.0 (5.6) | 404 | 33.9 (5.5) | 0.737 |
| **Maternal education** |  |  |  |  |  |
| Low | 56 | 10.4 | 91 | 22.7 | **<0.001** |
| Medium | 277 | 51.3 | 208 | 51.5 |  |
| High | 207 | 38.3 | 102 | 25.4 |  |
| **Paternal education** |  |  |  |  |  |
| Low | 158 | 30.2 | 143 | 37.0 | **0.037** |
| Medium | 224 | 42.7 | 163 | 42.1 |  |
| High | 142 | 27.1 | 81 | 20.9 |  |
| **Family origin** |  |  |  |  |  |
| Both Greek | 501 | 94.0 | 381 | 92.5 | 0.098 |
| Both foreign | 8 | 1.5 | 15 | 3.6 |  |
| Mixed | 24 | 4.5 | 16 | 3.9 |  |
| **Maternal working status** |  |  |  |  |  |
| Employed | 414 | 79.0 | 291 | 71.9 | **0.011** |
| Not working/Unemployed | 110 | 21.0 | 114 | 28.1 |  |
| **Paternal working status** |  |  |  |  |  |
| Employed | 526 | 99.4 | 406 | 98.5 | 0.164 |
| Unemployed | 3 | 0.6 | 6 | 1.5 |  |
| **Maternal marital status** |  |  |  |  |  |
| Married | 475 | 89.8 | 341 | 86.8 | 0.155 |
| Other | 54 | 10.2 | 52 | 13.2 |  |
| **Parity** |  |  |  |  |  |
| Nulliparous | 256 | 46.8 | 193 | 46.5 | 0.928 |
| Multiparous | 291 | 53.2 | 222 | 53.5 |  |
| **Area of living** |  |  |  |  |  |
| Urban | 377 | 79.0 | 255 | 75.2 | 0.199 |
| Rural | 100 | 21.0 | 84 | 24.7 |  |
| **Household income** (€, median, 25^th^ -75^th^) | 461 | 1040.7 (814.5, 1326.4) | 313 | 906.9 (689.1, 1186.3) | **<0.001** |
| **Perinatal characteristics** |  |  |  |  |  |
| **Sex** |  |  |  |  |  |
| Male | 287 | 52.1 | 224 | 52.7 | 0.848 |
| Female | 264 | 47.9 | 201 | 47.3 |  |
| **Preterm birth** |  |  |  |  |  |
| Yes | 66 | 12.3 | 61 | 15.5 | 0.218 |
| No | 471 | 87.7 | 344 | 84.9 |  |
| **Birth order** |  |  |  |  |  |
| First | 210 | 44.1 | 157 | 44.9 | 0.763 |
| Second | 182 | 38.2 | 126 | 36.0 |  |
| Third or more | 84 | 17.6 | 67 | 19.7 |  |
| **Mode of delivery** |  |  |  |  |  |
| Vaginal | 279 | 51.6 | 188 | 43.7 | **0.015** |
| C-section | 262 | 48.4 | 242 | 56.3 |  |
| **Smoking status during pregnancy** |  |  |  |  |  |
| Nonsmokers | 350 | 69.6 | 254 | 67.4 | 0.709 |
| Early pregnancy smokers | 65 | 12.9 | 49 | 13.0 |  |
| During pregnancy smokers | 88 | 17.5 | 74 | 19.6 |  |
| **BMI pre-pregnancy** (kg/m2) | 526 | 24.9 (5.2) | 389 | 24.3 (4.5) | **0.039** |
| **Birth weight** (kg) | 534 | 3.2 (0.4) | 422 | 3.1 (0.5) | **<0.001** |
| **Birth length** (cm) | 526 | 50.5 (2.3) | 400 | 50.0 (2.8) | **0.003** |
| **Birth head circumference** (cm) | 519 | 34.2 (1.4) | 388 | 33.9 (1.9) | **0.047** |
| **Gestational** **age** (weeks) | 537 | 38.2 (1.6) | 405 | 38.1 (1.7) | 0.364 |
| **Intensive Care Unit** |  |  |  |  |  |
| Yes | 91 | 18.3 | 75 | 19.7 | 0.583 |
| No | 407 | 81.7 | 305 | 80.3 |  |
| **Breastfeeding duration** (months) | 520 | 4.2 (4.1) | 391 | 3.6 (4.3) | **0.043** |

**Figure S1** Individual trajectories for (a) Internalizing, (b) Externalizing, and (c) ADHD symptoms

**Figure S2** Trajectory groups of internalizing (N=444), externalizing (N=446), and ADHD (N=443) symptoms from age 6 to 15 years (sensitivity analysis)

**Table S3** Data for selected trajectories of internalizing, externalizing, and ADHD symptoms from age 6 to 15 years (sensitivity analysis)

|  |  |  |  | Probability | | | |  | Average Posterior Probability | | | |  | Odds of Correct Classification | | | |
| --- | --- | --- | --- | --- | --- | --- | --- | --- | --- | --- | --- | --- | --- | --- | --- | --- | --- |
| Outcome | **Shape^a^** | **BIC** | **Entropy** | **G1** | **G2** | **G3** | **G4** |  | **G1** | **G2** | **G3** | **G4** |  | **G1** | **G2** | **G3** | **G4** |
| Internalizing symptoms | 0 2 2 2 | -1418.33 | 0.853 | 58.3 | 13.0 | 14.7 | 13.9 |  | 0.94 | 0.90 | 0.90 | 0.93 |  | 10.5 | 78.4 | 54.0 | 89.9 |
| Externalizing symptoms | 0 2 1 0 | -1382.35 | 0.864 | 62.6 | 15.7 | 12.8 | 8.9 |  | 0.96 | 0.86 | 0.88 | 0.90 |  | 14.9 | 32.3 | 50.9 | 88.8 |
| ADHD symptoms | 2 0 2 0 | -1368.44 | 0.892 | 12.3 | 61.8 | 16.0 | 9.9 |  | 0.95 | 0.96 | 0.94 | 0.95 |  | 148.2 | 11.9 | 99.3 | 156.7 |

*Abbreviations:* BIC: Bayesian Information Criterion; G: Group.

**^a^** Shape: 0 = zero-order, 1 = linear, 2 = quadratic.

**Table S4** Unadjusted associations of sociodemographic and perinatal factors with trajectory groups of **internalizing symptoms**, univariate analyses

|  |  | **Stable Low** | **High-decreasing** | **Low-increasing** | **Stable High** |  |
| --- | --- | --- | --- | --- | --- | --- |
|  | **Total N** | **Ν (%)** | **Ν (%)** | **Ν (%)** | **Ν (%)** | **p-value** |
| **Sociodemographic factors** |  |  |  |  |  |  |
| **Maternal age** [years; Mean (SD)] | 541 | 30.5 (4.4) | 28.7 (5.4) | 30.1 (4.9) | 28.5 (4.5) | **0.005** |
| **Paternal age** [years; Mean (SD)] | 524 | 34.5 (5.5) | 32.9 (6.0) | 33.1 (5.2) | 32.9 (6.5) | **0.026** |
| **Maternal education** | 536 |  |  |  |  | 0.124 |
| Low |  | 30 (54.5) | 10 (18.2) | 8 (14.5) | 7 (12.7) |  |
| Medium |  | 171 (62.4) | 32 (11.7) | 45 (16.4) | 26 (9.5) |  |
| High |  | 147 (71.0) | 17 (8.2) | 31 (15.0) | 12 (5.8) |  |
| **Paternal education** | 520 |  |  |  |  | 0.189 |
| Low |  | 93 (60.0) | 21 (13.5) | 21 (13.5) | 20 (12.9) |  |
| Medium |  | 150 (67.3) | 24 (10.8) | 34 (15.2) | 15 (6.7) |  |
| High |  | 97 (68.3) | 13 (9.2) | 24 (16.9) | 8 (5.6) |  |
| **Family origin** | 527 |  |  |  |  | 0.542 |
| Foreign/mixed |  | 19 (59.4) | 3 (9.4) | 8 (25.0) | 2 (6.3) |  |
| Greek |  | 323 (65.3) | 55 (11.1) | 76 (15.4) | 41 (8.3) |  |
| **Maternal working status (pregnancy)** | 520 |  |  |  |  | 0.390 |
| Unemployed |  | 67 (60.9) | 14 (12.7) | 16 (14.5) | 13 (11.8) |  |
| Working |  | 271 (66.1) | 43 (10.5) | 66 (16.1) | 30 (7.3) |  |
| **Paternal working status (pregnancy)** | 523 |  |  |  |  | 0.757 |
| Unemployed |  | 2 (66.7) | 0 (0.0) | 1 (33.3) | 0 (0.0) |  |
| Working |  | 338 (65.0) | 58 (11.2) | 81 (15.6) | 43 (8.3) |  |
| **Marital status** | 525 |  |  |  |  | 0.056 |
| Married |  | 312 (66.2) | 48 (10.2) | 71 (15.1) | 40 (8.5) |  |
| Other |  | 28 (51.9) | 10 (18.5) | 13 (24.1) | 3 (5.6) |  |
| **Parity** | 542 |  |  |  |  | 0.552 |
| Nulliparous |  | 156 (61.7) | 29 (11.5) | 44 (17.4) | 24 (9.5) |  |
| Multiparous |  | 195 (67.5) | 30 (10.4) | 42 (14.5) | 22 (7.6) |  |
| **Household income** (tertiles) | 546 |  |  |  |  | 0.337 |
| 1^st^ Tertile (<885 €) |  | 93 (60.8) | 22 (14.4) | 22 (14.4) | 16 (10.5) |  |
| 2^nd^ Tertile (885-1223 €) |  | 97 (64.2) | 14 (9.3) | 26 (17.2) | 14 (9.3) |  |
| 3^rd^ Tertile (1223 – 2180 €) |  | 110 (71.4) | 14 (9.1) | 24 (15.6) | 6 (3.9) |  |
| Missing |  | 54 (61.4) | 9 (10.2) | 15 (17.0) | 10 (11.4) |  |
| **Area of living** | 473 |  |  |  |  | 0.560 |
| Urban |  | 240 (64.0) | 39 (10.4) | 64 (17.1) | 32 (8.5) |  |
| Rural |  | 68 (69.4) | 10 (10.2) | 11 (11.2) | 9 (9.2) |  |
| **Perinatal factors** |  |  |  |  |  |  |
| **Smoking status during pregnancy** | 500 |  |  |  |  | 0.152 |
| Nonsmokers |  | 233 (67.0) | 37 (10.6) | 46 (13.2) | 32 (9.2) |  |
| Early pregnancy smokers |  | 42 (65.6) | 9 (14.1) | 11 (17.2) | 2 (3.1) |  |
| During pregnancy smokers |  | 50 (56.8) | 9 (10.2) | 21 (23.9) | 8 (9.1) |  |
| **BMI pre-pregnancy** [kg/m^2^; Mean (SD)] | 522 | 24.7 (5.2) | 24.9 (4.5) | 25.3 (5.5) | 25.9 (5.4) | 0.477 |
| **Sex** | 546 |  |  |  |  | **<0.001** |
| Male |  | 182 (63.9) | 43 (15.1) | 32 (11.2) | 28 (9.8) |  |
| Female |  | 172 (65.9) | 16 (6.1) | 55 (21.1) | 18 (6.9) |  |
| **Mode of delivery** | 536 |  |  |  |  | 0.607 |
| Vaginal |  | 176 (63.5) | 28 (10.1) | 48 (17.3) | 25 (9.0) |  |
| C-section |  | 172 (66.4) | 31 (12.0) | 37 (14.3) | 19 (7.3) |  |
| **Preterm birth** | 532 |  |  |  |  | 0.105 |
| No |  | 311 (66.5) | 49 (10.5) | 68 (14.5) | 40 (8.5) |  |
| Yes |  | 36 (56.3) | 10 (15.6) | 15 (23.4) | 3 (4.7) |  |
| **Birth order** | 472 |  |  |  |  | 0.552 |
| First |  | 123 (59.4) | 26 (12.6) | 39 (18.8) | 19 (9.2) |  |
| Second |  | 120 (65.9) | 20 (11.0) | 28 (15.4) | 14 (7.7) |  |
| Third or more |  | 60 (72.3) | 6 (7.2) | 11 (13.3) | 6 (7.2) |  |
| **Breastfeeding duration** [months; Median (IQR)] | 516 | 3.0 (1.0, 7.0) | 2.0 (0.8, 5.0) | 3.0 (1.0, 6.0) | 1.5 (0.1, 5.0) | 0.054 |
| **Birth weight** [grams; Mean (SD)] | 529 | 3205.7 (437.2) | 3149.9 (422.6) | 3134.9 (439.1) | 3339.5 (481.6) | 0.073 |
| **Birth length** [cm; Mean (SD)] | 522 | 50.5 (2.3) | 50.4 (2.2) | 50.1 (2.2) | 51.3 (2.2) | **0.044** |
| **Birth head circumference** [cm; Mean (SD)] | 515 | 34.1 (1.4) | 34.2 (1.3) | 34.1 (1.4) | 34.5 (1.5) | 0.457 |
| **Gestational age** [weeks; Mean (SD)] | 532 | 38.2 (1.5) | 38.0 (1.6) | 38.0 (1.8) | 38.5 (1.8) | 0.286 |
| **ICU attendance** | 495 |  |  |  |  | 0.174 |
| No |  | 262 (64.5) | 46 (11.3) | 60 (14.8) | 38 (9.4) |  |
| Yes |  | 62 (69.7) | 11 (12.4) | 14 (15.7) | 2 (2.2) |  |

*Abbreviations*: BMI: Body Mass Index; ICU: Intensive Care Unit.

Bold font indicates p < 0.05.

**Table S5** Unadjusted associations of sociodemographic and perinatal factors with trajectory groups of **externalizing symptoms**, univariate analyses

|  |  | **Stable Low** | **High-decreasing** | **Low-increasing** | **Stable High** |  |
| --- | --- | --- | --- | --- | --- | --- |
|  | **Total N** | **Ν (%)** | **Ν (%)** | **Ν (%)** | **Ν (%)** | **p-value** |
| **Sociodemographic factors** |  |  |  |  |  |  |
| **Maternal age** [years; Mean (SD)] | 540 | 30.7 (4.5) | 28.7 (5.4) | 29.7 (4.1) | 28.8 (5.0) | **0.001** |
| **Paternal age** [years; Mean (SD)] | 523 | 34.3 (5.6) | 33.4 (5.4) | 33.8 (6.1) | 32.8 (5.3) | 0.240 |
| **Maternal education** | 535 |  |  |  |  | **0.016** |
| Low |  | 28 (50.9) | 11 (20.0) | 12 (21.8) | 4 (7.3) |  |
| Medium |  | 151 (55.1) | 43 (15.7) | 48 (17.5) | 32 (11.7) |  |
| High |  | 142 (68.9) | 16 (7.8) | 31 (15.0) | 17 (8.3) |  |
| **Paternal education** | 519 |  |  |  |  | **<0.001** |
| Low |  | 74 (47.7) | 25 (16.1) | 29 (18.7) | 27 (17.4) |  |
| Medium |  | 141 (63.2) | 31 (13.9) | 37 (16.6) | 14 (6.3) |  |
| High |  | 99 (70.2) | 10 (7.1) | 23 (16.3) | 9 (6.4) |  |
| **Family origin** | 526 |  |  |  |  | 0.393 |
| Foreign/mixed |  | 18 (58.1) | 4 (12.9) | 8 (25.8) | 1 (3.2) |  |
| Greek |  | 300 (60.6) | 64 (12.9) | 81 (16.4) | 50 (10.1) |  |
| **Maternal working status (pregnancy)** | 519 |  |  |  |  | **0.020** |
| Unemployed |  | 59 (53.6) | 15 (13.6) | 17 (15.5) | 19 (17.3) |  |
| Working |  | 255 (62.3) | 52 (12.7) | 71 (17.4) | 31 (7.6) |  |
| **Paternal working status (pregnancy)** | 522 |  |  |  |  | 0.145 |
| Unemployed |  | 1 (33.3) | 0 (0.0) | 2 (66.7) | 0 (0.0) |  |
| Working |  | 314 (60.5) | 67 (12.9) | 87 (16.8) | 51 (9.8) |  |
| **Marital status** | 524 |  |  |  |  | 0.549 |
| Married |  | 287 (61.1) | 61 (13.0) | 79 (16.8) | 43 (9.1) |  |
| Other |  | 29 (53.7) | 7 (13.0) | 10 (18.5) | 8 (14.8) |  |
| **Parity** | 541 |  |  |  |  | 0.130 |
| Nulliparous |  | 143 (56.5) | 41 (16.2) | 46 (18.2) | 23 (9.1) |  |
| Multiparous |  | 183 (63.5) | 29 (10.1) | 46 (16.0) | 30 (10.4) |  |
| **Household income** (tertiles) | 545 |  |  |  |  | **0.014** |
| 1^st^ Tertile (<885 €) |  | 85 (55.6) | 23 (15.0) | 26 (17.0) | 19 (12.4) |  |
| 2^nd^ Tertile (885-1223 €) |  | 77 (51.3) | 26 (17.3) | 31 (20.7) | 16 (10.7) |  |
| 3^rd^ Tertile (1223 – 2180 €) |  | 114 (74.0) | 11 (7.1) | 20 (13.0) | 9 (5.8) |  |
| Missing |  | 54 (61.4) | 10 (11.4) | 15 (17.0) | 9 (10.2) |  |
| **Area of living** | 473 |  |  |  |  | 0.795 |
| Urban |  | 227 (60.5) | 48 (12.8) | 62 (16.5) | 38 (10.1) |  |
| Rural |  | 58 (59.2) | 12 (12.2) | 20 (20.4) | 8 (8.2) |  |
| **Perinatal factors** |  |  |  |  |  |  |
| **Smoking status during pregnancy** | 499 |  |  |  |  | **0.047** |
| Nonsmokers |  | 221 (63.7) | 37 (10.7) | 58 (16.7) | 31 (8.9) |  |
| Early pregnancy smokers |  | 39 (60.9) | 9 (14.1) | 12 (18.8) | 4 (6.3) |  |
| During pregnancy smokers |  | 43 (48.9) | 19 (21.6) | 13 (14.8) | 13 (14.8) |  |
| **BMI pre-pregnancy** [kg/m^2^; Mean (SD)] | 521 | 24.5 (4.9) | 25.4 (5.2) | 25.7 (5.9) | 25.9 (5.5) | 0.074 |
| **Sex** | 545 |  |  |  |  | **<0.001** |
| Male |  | 151 (53.0) | 47 (16.5) | 50 (17.5) | 37 (13.0) |  |
| Female |  | 179 (68.8) | 23 (8.8) | 42 (16.2) | 16 (6.2) |  |
| **Mode of delivery** | 535 |  |  |  |  | 0.689 |
| Vaginal |  | 161 (58.1) | 39 (14.1) | 49 (17.7) | 20 (10.1) |  |
| C-section |  | 163 (63.2) | 31 (12.0) | 40 (15.5) | 24 (9.3) |  |
| **Preterm birth** | 531 |  |  |  |  | 0.145 |
| No |  | 290 (62.1) | 59 (12.6) | 73 (15.6) | 45 (9.6) |  |
| Yes |  | 32 (50.0) | 11 (17.2) | 16 (25.0) | 5 (7.8) |  |
| **Birth order** | 471 |  |  |  |  | 0.526 |
| First |  | 120 (58.0) | 30 (14.5) | 34 (16.4) | 23 (11.1) |  |
| Second |  | 111 (61.0) | 21 (11.5) | 34 (18.7) | 16 (8.8) |  |
| Third or more |  | 56 (68.3) | 6 (7.3) | 11 (13.4) | 9 (11.0) |  |
| **Breastfeeding duration** [months; Median (IQR)] | 515 | 3.0 (1.0, 7.0) | 1.0 (0.5, 5.0) | 3.0 (2.0, 6.0) | 3.0 (1.0, 5.0) | **0.014** |
| **Birth weight** [grams; Mean (SD)] | 528 | 3191.5 (437.2) | 3179.9 (428.6) | 3204.3 (396.1) | 3281.0 (554.8) | 0.583 |
| **Birth length** [cm; Mean (SD)] | 521 | 50.4 (2.2) | 50.5 (2.5) | 50.4 (2.3) | 50.7 (2.6) | 0.867 |
| **Birth head circumference** [cm; Mean (SD)] | 514 | 34.1 (1.4) | 34.2 (1.6) | 34.2 (1.1) | 34.4 (1.4) | 0.562 |
| **Gestational age** [weeks; Mean (SD)] | 531 | 38.2 (1.5) | 38.3 (1.8) | 38.0 (1.6) | 38.3 (2.0) | 0.736 |
| **ICU attendance** | 494 |  |  |  |  | 0.450 |
| No |  | 254 (62.7) | 48 (11.9) | 64 (15.8) | 39 (9.6) |  |
| Yes |  | 49 (55.1) | 15 (16.9) | 17 (19.1) | 8 (9.0) |  |

*Abbreviations*: BMI: Body Mass Index; ICU: Intensive Care Unit.

Bold font indicates p < 0.05.

**Table S6** Unadjusted associations of sociodemographic and perinatal factors with trajectory groups of **ADHD symptoms**, univariate analyses

|  |  | **Stable Low** | **High-decreasing** | **Low-increasing** | **Stable High** |  |
| --- | --- | --- | --- | --- | --- | --- |
|  | **Total N** | **Ν (%)** | **Ν (%)** | **Ν (%)** | **Ν (%)** | **p-value** |
| **Sociodemographic factors** |  |  |  |  |  |  |
| **Maternal age** [years; Mean (SD)] | 544 | 30.7 (4.3) | 28.0 (4.9) | 29.9 (4.8) | 29.7 (5.5) | **<0.001** |
| **Paternal age** [years; Mean (SD)] | 526 | 34.3 (5.4) | 32.6 (5.0) | 34.6 (6.7) | 33.8 (5.9) | 0.067 |
| **Maternal education** | 538 |  |  |  |  | 0.135 |
| Low |  | 29 (51.8) | 13 (23.2) | 10 (17.9) | 4 (7.1) |  |
| Medium |  | 157 (56.9) | 51 (18.5) | 41 (14.9) | 27 (9.8) |  |
| High |  | 136 (66.0) | 22 (10.7) | 28 (13.6) | 20 (9.7) |  |
| **Paternal education** | 522 |  |  |  |  | **0.002** |
| Low |  | 83 (52.5) | 40 (25.3) | 17 (10.8) | 18 (11.4) |  |
| Medium |  | 133 (59.6) | 31 (13.9) | 41 (18.4) | 18 (8.1) |  |
| High |  | 97 (68.8) | 14 (9.9) | 18 (12.8) | 12 (8.5) |  |
| **Family origin** | 529 |  |  |  |  | 0.374 |
| Foreign/mixed |  | 16 (50.0) | 5 (15.6) | 8 (25.0) | 3 (9.4) |  |
| Greek |  | 301 (60.6) | 80 (16.1) | 69 (13.9) | 47 (9.5) |  |
| **Maternal working status (pregnancy)** | 526 |  |  |  |  | **0.022** |
| Unemployed |  | 60 (54.5) | 28 (25.5) | 14 (12.7) | 8 (7.3) |  |
| Working |  | 254 (61.7) | 55 (13.3) | 63 (15.3) | 40 (9.7) |  |
| **Paternal working status (pregnancy)** | 525 |  |  |  |  | 0.078 |
| Unemployed |  | 1 (33.3) | 0 (0.0) | 2 (66.7) | 0 (0.0) |  |
| Working |  | 313 (60.0) | 85 (16.3) | 74 (14.2) | 50 (9.6) |  |
| **Marital status** | 527 |  |  |  |  | 0.105 |
| Married |  | 291 (61.5) | 75 (15.9) | 63 (13.3) | 44 (9.3) |  |
| Other |  | 25 (46.3) | 10 (18.5) | 13 (24.1) | 6 (11.1) |  |
| **Parity** | 545 |  |  |  |  | 0.235 |
| Nulliparous |  | 145 (56.9) | 47 (18.4) | 35 (13.7) | 28 (11.0) |  |
| Multiparous |  | 182 (62.8) | 39 (13.4) | 45 (15.5) | 24 (8.3) |  |
| **Household income** (tertiles) | 549 |  |  |  |  | **0.014** |
| 1^st^ Tertile (<885 €) |  | 84 (54.9) | 35 (22.9) | 18 (11.8) | 16 (10.5) |  |
| 2^nd^ Tertile (885-1223 €) |  | 84 (54.5) | 24 (15.6) | 31 (20.1) | 15 (9.7) |  |
| 3^rd^ Tertile (1223 – 2180 €) |  | 110 (71.9) | 15 (9.8) | 16 (10.5) | 12 (7.8) |  |
| Missing |  | 52 (58.4) | 12 (13.5) | 15 (16.9) | 10 (11.2) |  |
| **Area of living** | 475 |  |  |  |  | 0.051 |
| Urban |  | 226 (60.3) | 53 (14.1) | 60 (16.0) | 36 (9.6) |  |
| Rural |  | 62 (62.0) | 23 (23.0) | 10 (10.0) | 5 (5.0) |  |
| **Perinatal factors** |  |  |  |  |  |  |
| **Smoking status during pregnancy** | 501 |  |  |  |  | 0.181 |
| Nonsmokers |  | 219 (62.9) | 51 (14.7) | 51 (14.7) | 27 (7.8) |  |
| Early pregnancy smokers |  | 38 (58.5) | 13 (20.0) | 7 (10.8) | 7 (10.8) |  |
| During pregnancy smokers |  | 45 (51.1) | 17 (19.3) | 12 (13.6) | 14 (15.9) |  |
| **BMI pre-pregnancy** [kg/m^2^; Mean (SD)] | 524 | 24.8 (5.2) | 25.3 (5.1) | 24.5 (5.2) | 25.5 (4.9) | 0.657 |
| **Sex** | 549 |  |  |  |  | **0.001** |
| Male |  | 150 (52.4) | 53 (18.5) | 46 (16.1) | 37 (12.9) |  |
| Female |  | 180 (68.4) | 33 (12.5) | 34 (12.9) | 16 (6.1) |  |
| **Mode of delivery** | 539 |  |  |  |  | 0.399 |
| Vaginal |  | 166 (59.5) | 49 (17.6) | 42 (15.1) | 22 (7.9) |  |
| C-section |  | 157 (60.4) | 37 (14.2) | 36 (13.8) | 30 (11.5) |  |
| **Preterm birth** | 535 |  |  |  |  | 0.140 |
| No |  | 289 (61.5) | 72 (15.3) | 64 (13.6) | 45 (9.6) |  |
| Yes |  | 32 (49.2) | 14 (21.5) | 14 (21.5) | 5 (7.7) |  |
| **Birth order** | 474 |  |  |  |  | 0.240 |
| First |  | 118 (56.5) | 42 (20.1) | 25 (12.0) | 24 (11.5) |  |
| Second |  | 109 (60.2) | 28 (15.5) | 30 (16.6) | 14 (7.7) |  |
| Third or more |  | 56 (66.7) | 9 (10.7) | 13 (15.5) | 6 (7.1) |  |
| **Breastfeeding duration** [months; Median (IQR)] | 518 | 3.0 (1.0, 7.0) | 2.0 (0.5, 3.0) | 3.0 (1.0, 7.0) | 2.0 (1.0, 6.0) | **0.001** |
| **Birth weight** [grams; Mean (SD)] | 532 | 3205.0 (423.7) | 3155.6 (452.5) | 3178.9 (455.0) | 3281.3 (547.8) | 0.433 |
| **Birth length** [cm; Mean (SD)] | 524 | 50.5 (2.1) | 50.4 (2.7) | 50.2 (2.5) | 51.0 (2.4) | 0.315 |
| **Birth head circumference** [cm; Mean (SD)] | 517 | 34.1 (1.4) | 34.4 (1.4) | 34.2 (1.3) | 34.1 (1.7) | 0.327 |
| **Gestational age** [weeks; Mean (SD)] | 535 | 38.2 (1.5) | 38.1 (1.7) | 38.0 (1.7) | 38.5 (2.0) | 0.320 |
| **ICU attendance** | 496 |  |  |  |  | 0.487 |
| No |  | 251 (61.8) | 61 (15.0) | 58 (14.3) | 36 (8.9) |  |
| Yes |  | 49 (54.4) | 19 (21.1) | 14 (15.6) | 8 (8.9) |  |

*Abbreviations*: BMI: Body Mass Index; ICU: Intensive Care Unit.

Bold font indicates p < 0.05.

**Table S7** Adjusted associations of sociodemographic and perinatal factors with trajectory groups of internalizing, externalizing, and ADHD symptoms, multinomial logistic regression models with varying reference groups

|  | **Ref: High-decreasing** | | | **Ref: Low-increasing** | | | **Ref: Stable High** | | |
| --- | --- | --- | --- | --- | --- | --- | --- | --- | --- |
|  | **Stable Low** | **Low-increasing** | **Stable High** | **Stable Low** | **High-decreasing** | **Stable High** | **Stable Low** | **High-decreasing** | **Low-increasing** |
|  | **RRR (95% CI)** | **RRR (95% CI)** | **RRR (95% CI)** | **RRR (95% CI)** | **RRR (95% CI)** | **RRR (95% CI)** | **RRR (95% CI)** | **RRR (95% CI)** | **RRR (95% CI)** |
| **Internalizing symptoms** | | | | | | | | | |
| **Maternal age** (years) |  |  |  |  |  |  |  |  |  |
| Spline 1* | **1.17 (1.08, 1.26)** | **1.19 (1.07, 1.32)** | 1.07 (0.96, 1.19) | 0.98 (0.9, 1.07) | **0.84 (0.76, 0.93)** | 0.90 (0.80, 1.01) | 1.09 (0.99, 1.20) | 0.94 (0.84, 1.04) | 1.11 (0.99, 1.25) |
| Spline 2** | 0.95 (0.86, 1.05) | 1.02 (0.89, 1.17) | 0.94 (0.81, 1.09) | 0.93 (0.84, 1.04) | 0.98 (0.86, 1.13) | 0.92 (0.79, 1.07) | 1.01 (0.89, 1.16) | 1.07 (0.92, 1.23) | 1.09 (0.94, 1.26) |
| **Paternal age** (years) | 1.01 (0.97, 1.06) | 0.94 (0.88, 1.01) | 0.98 (0.91, 1.06) | **1.07 (1.02, 1.13)** | 1.06 (0.99, 1.13) | 1.04 (0.96, 1.12) | 1.03 (0.96, 1.11) | 1.02 (0.95, 1.1) | 0.96 (0.89, 1.04) |
| **Sex** |  |  |  |  |  |  |  |  |  |
| Female (ref. Male) | **2.18 (1.36, 3.48)** | **3.62 (2.05, 6.38)** | 1.65 (0.85, 3.20) | **0.60 (0.40, 0.91)** | **0.28 (0.16, 0.49)** | **0.46 (0.24, 0.86)** | 1.32 (0.74, 2.37) | 0.61 (0.31, 1.17) | **2.19 (1.16, 4.13)** |
| **Birth length** (per 1cm increase) | 1.01 (0.92, 1.11) | 1.00 (0.88, 1.13) | 1.13 (0.99, 1.29) | 1.02 (0.92, 1.12) | 1.00 (0.89, 1.13) | 1.13 (0.98, 1.30) | 0.90 (0.79, 1.02) | 0.89 (0.78, 1.01) | 0.88 (0.77, 1.02) |
| **Externalizing symptoms** | | | | | | | | | |
| **Maternal age** (years) |  |  |  |  |  |  |  |  |  |
| Spline 1* | **1.30 (1.09, 1.55)** | **1.45 (1.12, 1.88)** | 1.08 (0.87, 1.34) | 0.89 (0.70, 1.15) | **0.69 (0.53, 0.89)** | **0.74 (0.56, 0.98)** | 1.20 (0.97, 1.49) | 0.92 (0.74, 1.15) | **1.34 (1.02, 1.77)** |
| Spline 2** | 0.99 (0.93, 1.06) | 0.93 (0.87, 1.01) | 0.98 (0.89, 1.08) | **1.07 (1.00, 1.13)** | 1.07 (0.99, 1.16) | 1.05 (0.96, 1.15) | 1.01 (0.94, 1.1) | 1.02 (0.93, 1.12) | 0.95 (0.87, 1.04) |
| **Maternal education** |  |  |  |  |  |  |  |  |  |
| Medium (ref.: Low) | 1.26 (0.61, 2.58) | 1.01 (0.45, 2.25) | 2.09 (0.69, 6.32) | 1.25 (0.63, 2.49) | 0.99 (0.45, 2.21) | 2.07 (0.67, 6.39) | 0.60 (0.20, 1.78) | 0.48 (0.16, 1.45) | 0.48 (0.16, 1.49) |
| High (ref.: Low) | 1.82 (0.77, 4.30) | 1.37 (0.49, 3.79) | **4.32 (1.14, 16.38)** | 1.33 (0.55, 3.24) | 0.73 (0.26, 2.03) | 3.16 (0.8, 12.41) | 0.42 (0.12, 1.53) | **0.23 (0.06, 0.88)** | 0.32 (0.08, 1.25) |
| **Paternal education** |  |  |  |  |  |  |  |  |  |
| Medium (ref.: Low) | 1.16 (0.67, 2.00) | 0.90 (0.47, 1.71) | **0.42 (0.20, 0.87)** | 1.29 (0.77, 2.17) | 1.12 (0.59, 2.13) | **0.47 (0.23, 0.95)** | **2.77 (1.43, 5.39)** | **2.4 (1.15, 5.01)** | **2.15 (1.05, 4.39)** |
| High (ref.: Low) | 1.47 (0.67, 3.21) | 1.30 (0.52, 3.28) | 0.66 (0.21, 2.03) | 1.13 (0.56, 2.26) | 0.77 (0.30, 1.93) | 0.50 (0.18, 1.45) | 2.23 (0.84, 5.90) | 1.52 (0.49, 4.69) | 1.98 (0.69, 5.69) |
| **Maternal working status** (pregnancy) |  |  |  |  |  |  |  |  |  |
| Working (ref.: Unemployed) | 1.04 (0.56, 1.93) | 1.28 (0.62, 2.64) | 0.55 (0.23, 1.33) | 0.81 (0.44, 1.49) | 0.78 (0.38, 1.62) | 0.43 (0.18, 1.04) | 1.88 (0.82, 4.28) | 1.81 (0.75, 4.37) | 2.31 (0.96, 5.55) |
| **Household income** (tertiles) |  |  |  |  |  |  |  |  |  |
| 2^nd^ Tertile (ref.: 1^st^) | 0.69 (0.36, 1.35) | 0.83 (0.38, 1.82) | 1.06 (0.41, 2.75) | 0.83 (0.44, 1.57) | 1.20 (0.55, 2.61) | 1.27 (0.51, 3.16) | 0.65 (0.27, 1.58) | 0.94 (0.36, 2.43) | 0.78 (0.32, 1.95) |
| 3^rd^ Tertile (ref.: 1^st^) | 1.40 (0.59, 3.32) | 0.97 (0.33, 2.80) | 0.90 (0.25, 3.22) | 1.45 (0.60, 3.50) | 1.03 (0.36, 2.99) | 0.93 (0.26, 3.32) | 1.55 (0.49, 4.96) | 1.11 (0.31, 3.94) | 1.07 (0.30, 3.82) |
| **Smoking status during pregnancy** |  |  |  |  |  |  |  |  |  |
| Early pregnancy smokers  (ref.: Nonsmokers) | 0.90 (0.44, 1.83) | 0.97 (0.42, 2.25) | 0.87 (0.29, 2.64) | 0.93 (0.5, 1.72) | 1.03 (0.44, 2.37) | 0.89 (0.31, 2.61) | 1.04 (0.38, 2.84) | 1.15 (0.38, 3.47) | 1.12 (0.38, 3.26) |
| During pregnancy smokers (ref.: Nonsmokers) | 0.57 (0.31, 1.04) | 0.62 (0.3, 1.28) | 1.29 (0.56, 2.95) | 0.91 (0.49, 1.71) | 1.62 (0.78, 3.34) | 2.08 (0.91, 4.75) | **0.44 (0.20, 0.95)** | 0.78 (0.34, 1.78) | 0.48 (0.21, 1.10) |
| **Sex** |  |  |  |  |  |  |  |  |  |
| Female (ref. Male) | **2.37 (1.47, 3.83)** | **1.76 (1.02, 3.06)** | 0.83 (0.41, 1.65) | 1.35 (0.89, 2.03) | **0.57 (0.33, 0.98)** | **0.47 (0.24, 0.9)** | **2.87 (1.56, 5.29)** | 1.21 (0.61, 2.42) | **2.13 (1.11, 4.10)** |
| **Breastfeeding duration** (months) | 1.02 (0.95, 1.10) | 1.03 (0.94, 1.12) | 1.01 (0.92, 1.11) | 1.00 (0.95, 1.05) | 0.98 (0.90, 1.06) | 0.99 (0.91, 1.07) | 1.01 (0.94, 1.09) | 0.99 (0.90, 1.08) | 1.01 (0.94, 1.09) |
| **ADHD symptoms** | | | | | | | | | |
| **Maternal age** (years) |  |  |  |  |  |  |  |  |  |
| Spline 1* | **1.25 (1.04, 1.50)** | 1.12 (0.90, 1.38) | 0.89 (0.73, 1.08) | 1.12 (0.89, 1.40) | 0.90 (0.72, 1.11) | 0.79 (0.62, 1.02) | **1.41 (1.13, 1.76)** | 1.13 (0.93, 1.37) | 1.26 (0.98, 1.61) |
| Spline 2** | 1.06 (0.99, 1.13) | 1.02 (0.95, 1.11) | **1.12 (1.03, 1.21)** | 1.03 (0.97, 1.10) | 0.98 (0.9, 1.06) | **1.09 (1.00, 1.18)** | 0.95 (0.88, 1.02) | **0.90 (0.83, 0.97)** | **0.92 (0.85, 1.00)** |
| **Maternal education** |  |  |  |  |  |  |  |  |  |
| Medium (ref.: Low) | 1.33 (0.68, 2.62) | 0.87 (0.38, 2.03) | 1.60 (0.54, 4.77) | 1.53 (0.72, 3.24) | 1.14 (0.49, 2.65) | 1.83 (0.58, 5.71) | 0.83 (0.28, 2.52) | 0.63 (0.21, 1.86) | 0.55 (0.18, 1.71) |
| High (ref.: Low) | 1.13 (0.50, 2.57) | 0.85 (0.30, 2.40) | 1.97 (0.53, 7.41) | 1.33 (0.55, 3.24) | 1.18 (0.42, 3.35) | 2.33 (0.60, 9.07) | 0.57 (0.15, 2.12) | 0.51 (0.13, 1.90) | 0.43 (0.11, 1.67) |
| **Paternal education** |  |  |  |  |  |  |  |  |  |
| Medium (ref.: Low) | 1.41 (0.88, 2.27) | **2.22 (1.20, 4.13)** | 1.00 (0.50, 2.01) | 0.63 (0.36, 1.11) | **0.45 (0.24, 0.83)** | **0.45 (0.21, 0.95)** | 1.41 (0.71, 2.81) | 1.00 (0.50, 2.01) | **2.22 (1.05, 4.69)** |
| High (ref.: Low) | 1.45 (0.71, 2.96) | 2.25 (0.95, 5.34) | 0.90 (0.29, 2.77) | 0.64 (0.32, 1.31) | 0.44 (0.19, 1.05) | 0.40 (0.13, 1.20) | 1.61 (0.59, 4.45) | 1.11 (0.36, 3.44) | 2.51 (0.84, 7.51) |
| **Maternal working status** (pregnancy) |  |  |  |  |  |  |  |  |  |
| Working (ref.: Unemployed) | 1.37 (0.78, 2.41) | 1.62 (0.80, 3.26) | 2.28 (0.89, 5.87) | 0.85 (0.45, 1.58) | 0.62 (0.31, 1.24) | 1.41 (0.51, 3.88) | 0.60 (0.24, 1.53) | 0.44 (0.17, 1.13) | 0.71 (0.26, 1.95) |
| **Household income** (tertiles) |  |  |  |  |  |  |  |  |  |
| 2^nd^ Tertile (ref.: 1^st^) | 0.94 (0.52, 1.70) | 1.18 (0.54, 2.55) | 0.85 (0.34, 2.11) | 0.80 (0.40, 1.58) | 0.85 (0.39, 1.84) | 0.72 (0.27, 1.91) | 1.10 (0.45, 2.69) | 1.18 (0.47, 2.92) | 1.39 (0.52, 3.66) |
| 3^rd^ Tertile (ref.: 1^st^) | 1.48 (0.61, 3.64) | 0.87 (0.29, 2.62) | 0.62 (0.18, 2.17) | 1.70 (0.72, 4.00) | 1.15 (0.38, 3.45) | 0.71 (0.21, 2.35) | 2.40 (0.76, 7.57) | 1.62 (0.46, 5.68) | 1.41 (0.43, 4.66) |
| **Sex** |  |  |  |  |  |  |  |  |  |
| Female (ref. Male) | **1.92 (1.26, 2.91)** | 1.13 (0.67, 1.91) | 0.64 (0.34, 1.21) | **1.69 (1.10, 2.60)** | 0.88 (0.52, 1.49) | 0.57 (0.30, 1.08) | **2.99 (1.66, 5.39)** | 1.56 (0.83, 2.94) | 1.77 (0.93, 3.37) |
| **Breastfeeding duration** (months) | **1.09 (1.02, 1.16)** | 1.07 (0.99, 1.16) | 1.05 (0.95, 1.15) | 1.02 (0.96, 1.08) | 0.94 (0.86, 1.01) | 0.98 (0.90, 1.07) | 1.04 (0.96, 1.13) | 0.96 (0.87, 1.05) | 1.02 (0.94, 1.12) |

Bold font indicates p < 0.05.

*Spline 1: maternal age≤30 for internalizing symptoms and ≤25 for externalizing and ADHD symptoms.

**Spline 2: maternal age>30 for internalizing symptoms and >25 for externalizing and ADHD symptoms.

**Table S8** Adjusted associations of sociodemographic and perinatal factors with trajectory groups of internalizing, externalizing, and ADHD symptoms separately for boys and girls

|  | **Boys** | **Girls** | **p-interaction^#^** |
| --- | --- | --- | --- |
|  | **RRR (95% CI)** | **RRR (95% CI)** |  |
| **Internalizing symptoms** | | | |
| **High-decreasing** |  |  |  |
| **Maternal age** (years) |  |  |  |
| Spline 1* | **0.87 (0.78, 0.96)** | **0.83 (0.75, 0.92)** | 0.620 |
| Spline 2** | 1.11 (0.97, 1.27) | 0.93 (0.82, 1.06) | 0.218 |
| **Paternal age** (years) | 0.96 (0.91, 1.02) | 1.04 (0.95, 1.13) | 0.694 |
| **Birth length** (per 1cm increase) | 0.98 (0.86, 1.11) | 0.99 (0.86, 1.15) | 0.890 |
| **Low-increasing** |  |  |  |
| **Maternal age** (years) |  |  |  |
| Spline 1* | 1.06 (0.94, 1.18) | 0.99 (0.88, 1.13) | 0.628 |
| Spline 2** | 1.08 (0.9, 1.3) | 1.05 (0.92, 1.19) | 0.862 |
| **Paternal age** (years) | **0.91 (0.83, 1)** | 0.95 (0.89, 1.01) | 0.753 |
| **Birth length** (per 1cm increase) | 1 (0.86, 1.17) | 0.97 (0.86, 1.1) | 0.728 |
| **Stable High** |  |  |  |
| **Maternal age** (years) |  |  |  |
| Spline 1* | 0.92 (0.81, 1.04) | 0.92 (0.77, 1.09) | 0.637 |
| Spline 2** | 1.02 (0.85, 1.21) | 0.95 (0.78, 1.16) | 0.866 |
| **Paternal age** (years) | 0.94 (0.84, 1.05) | 1 (0.91, 1.11) | 0.419 |
| **Birth length** (per 1cm increase) | 1.1 (0.94, 1.3) | 1.13 (0.92, 1.37) | 0.872 |
| **Externalizing symptoms** | | | |
| **High-decreasing** |  |  |  |
| **Maternal age** (years) |  |  |  |
| Spline 1* | **0.67 (0.5, 0.89)** | 0.86 (0.66, 1.12) | 0.153 |
| Spline 2** | 1.01 (0.92, 1.11) | 1.01 (0.91, 1.12) | 0.595 |
| **Maternal education** |  |  |  |
| Medium (ref.: Low) | 0.57 (0.23, 1.41) | 1.76 (0.5, 6.27) | 0.158 |
| High (ref.: Low) | 0.44 (0.13, 1.44) | 0.88 (0.2, 3.78) | 0.329 |
| **Paternal education** |  |  |  |
| Medium (ref.: Low) | 0.69 (0.35, 1.37) | 1.18 (0.45, 3.07) | 0.276 |
| High (ref.: Low) | 0.72 (0.28, 1.85) | 0.6 (0.14, 2.59) | 0.966 |
| **Maternal working status** (pregnancy) |  |  |  |
| Working (ref.: Unemployed) | 1.19 (0.54, 2.63) | 0.58 (0.2, 1.65) | 0.528 |
| **Household income** (tertiles) |  |  |  |
| 2^nd^ Tertile (ref.: 1^st^) | 1.52 (0.66, 3.48) | 1.47 (0.44, 4.94) | 0.890 |
| 3^rd^ Tertile (ref.: 1^st^) | 0.63 (0.19, 2.09) | 1.04 (0.27, 4.01) | 0.645 |
| **Smoking status during pregnancy** |  |  |  |
| Early pregnancy smokers (ref.: Nonsmokers) | 1.24 (0.52, 2.95) | 1.04 (0.29, 3.72) | 0.593 |
| During pregnancy smokers (ref.: Nonsmokers) | 1.59 (0.69, 3.67) | 2.38 (0.92, 6.13) | 0.724 |
| **Breastfeeding duration** (months) | 0.97 (0.88, 1.06) | 1 (0.88, 1.12) | 0.665 |
| **Low-increasing** |  |  |  |
| **Maternal age** (years) |  |  |  |
| Spline 1* | 0.93 (0.62, 1.39) | 1.28 (0.95, 1.74) | 0.237 |
| Spline 2** | 0.95 (0.87, 1.05) | **0.91 (0.84, 0.99)** | 0.765 |
| **Maternal education** |  |  |  |
| Medium (ref.: Low) | 1.12 (0.37, 3.33) | 0.58 (0.22, 1.56) | 0.495 |
| High (ref.: Low) | 1.09 (0.28, 4.15) | 0.51 (0.13, 1.93) | 0.337 |
| **Paternal education** |  |  |  |
| Medium (ref.: Low) | 0.6 (0.29, 1.25) | 1.07 (0.49, 2.36) | 0.362 |
| High (ref.: Low) | 0.77 (0.28, 2.09) | 1.11 (0.42, 2.92) | 0.947 |
| **Maternal working status** (pregnancy) |  |  |  |
| Working (ref.: Unemployed) | 1.19 (0.53, 2.68) | 1.45 (0.56, 3.76) | 0.888 |
| **Household income** (tertiles) |  |  |  |
| 2^nd^ Tertile (ref.: 1^st^) | 1.18 (0.49, 2.81) | 1.31 (0.52, 3.34) | 0.740 |
| 3^rd^ Tertile (ref.: 1^st^) | 0.8 (0.23, 2.73) | 0.58 (0.16, 2.13) | 0.561 |
| **Smoking status during pregnancy** |  |  |  |
| Early pregnancy smokers (ref.: Nonsmokers) | 1.47 (0.66, 3.26) | 0.67 (0.23, 1.98) | 0.235 |
| During pregnancy smokers (ref.: Nonsmokers) | 0.96 (0.38, 2.47) | 1.27 (0.56, 2.87) | 0.609 |
| **Breastfeeding duration** (months) | 1 (0.94, 1.08) | 1.01 (0.93, 1.1) | 0.952 |
| **Stable High** |  |  |  |
| **Maternal age** (years) |  |  |  |
| Spline 1* | 0.79 (0.56, 1.12) | 0.8 (0.58, 1.09) | 0.346 |
| Spline 2** | 0.94 (0.85, 1.04) | 1.07 (0.95, 1.21) | 0.134 |
| **Maternal education** |  |  |  |
| Medium (ref.: Low) | 1.43 (0.34, 5.99) | 1.8 (0.24, 13.72) | 0.949 |
| High (ref.: Low) | 1.12 (0.21, 6.03) | 6.12 (0.6, 62.78) | 0.388 |
| **Paternal education** |  |  |  |
| Medium (ref.: Low) | **0.37 (0.17, 0.83)** | **0.27 (0.09, 0.84)** | 0.975 |
| High (ref.: Low) | 0.73 (0.25, 2.2) | 0.19 (0.03, 1.37) | 0.585 |
| **Maternal working status** (pregnancy) |  |  |  |
| Working (ref.: Unemployed) | 0.44 (0.17, 1.16) | 0.78 (0.18, 3.29) | 0.402 |
| **Household income** (tertiles) |  |  |  |
| 2^nd^ Tertile (ref.: 1^st^) | 1.47 (0.51, 4.2) | 1.56 (0.28, 8.69) | 0.288 |
| 3^rd^ Tertile (ref.: 1^st^) | 1 (0.25, 4.06) | 0.39 (0.03, 4.67) | 0.801 |
| **Smoking status during pregnancy** |  |  |  |
| Early pregnancy smokers (ref.: Nonsmokers) | 0.49 (0.1, 2.27) | 2.02 (0.46, 8.83) | 0.213 |
| During pregnancy smokers (ref.: Nonsmokers) | **2.74 (1.06, 7.06)** | 1.81 (0.48, 6.89) | 0.547 |
| **Breastfeeding duration** (months) | 1 (0.91, 1.1) | 0.97 (0.87, 1.09) | 0.821 |
| **ADHD symptoms** | | | |
| **High-decreasing** |  |  |  |
| **Maternal age** (years) |  |  |  |
| Spline 1* | 0.77 (0.56, 1.05) | 0.85 (0.68, 1.06) | 0.844 |
| Spline 2** | 0.94 (0.86, 1.03) | 0.95 (0.87, 1.04) | 0.704 |
| **Maternal education** |  |  |  |
| Medium (ref.: Low) | 0.61 (0.24, 1.53) | 0.94 (0.33, 2.67) | 0.665 |
| High (ref.: Low) | 0.48 (0.14, 1.58) | 1.82 (0.58, 5.74) | 0.830 |
| **Paternal education** |  |  |  |
| Medium (ref.: Low) | 0.77 (0.4, 1.46) | 0.62 (0.3, 1.25) | 0.592 |
| High (ref.: Low) | 0.98 (0.38, 2.48) | 0.46 (0.13, 1.6) | 0.091 |
| **Maternal working status** (pregnancy) |  |  |  |
| Working (ref.: Unemployed) | 0.56 (0.28, 1.13) | 1.07 (0.41, 2.77) | 0.702 |
| **Household income** (tertiles) |  |  |  |
| 2^nd^ Tertile (ref.: 1^st^) | 1.24 (0.59, 2.6) | 0.81 (0.3, 2.18) | 0.868 |
| 3^rd^ Tertile (ref.: 1^st^) | 1.38 (0.46, 4.12) | 0.24 (0.05, 1.06) | 0.074 |
| **Breastfeeding duration** (months) | 0.94 (0.86, 1.03) | **0.88 (0.79, 0.97)** | 0.148 |
| **Low-increasing** |  |  |  |
| **Maternal age** (years) |  |  |  |
| Spline 1* | 0.78 (0.54, 1.12) | 1.05 (0.82, 1.34) | 0.286 |
| Spline 2** | 0.98 (0.9, 1.07) | 0.95 (0.86, 1.05) | 0.751 |
| **Maternal education** |  |  |  |
| Medium (ref.: Low) | 0.74 (0.24, 2.21) | 0.66 (0.21, 2.09) | 0.806 |
| High (ref.: Low) | 0.72 (0.19, 2.7) | 0.82 (0.22, 3.06) | 0.885 |
| **Paternal education** |  |  |  |
| Medium (ref.: Low) | 1.81 (0.87, 3.76) | 1.25 (0.53, 2.95) | 0.485 |
| High (ref.: Low) | 1.87 (0.74, 4.73) | 1.19 (0.39, 3.59) | 0.469 |
| **Maternal working status** (pregnancy) |  |  |  |
| Working (ref.: Unemployed) | 1.55 (0.68, 3.53) | 0.77 (0.27, 2.25) | 0.292 |
| **Household income** (tertiles) |  |  |  |
| 2^nd^ Tertile (ref.: 1^st^) | 1.15 (0.5, 2.65) | 1.56 (0.46, 5.24) | 0.850 |
| 3^rd^ Tertile (ref.: 1^st^) | 0.57 (0.18, 1.78) | 0.72 (0.17, 3.11) | 0.870 |
| **Breastfeeding duration** (months) | 1 (0.93, 1.07) | 0.97 (0.87, 1.07) | 0.669 |
| **Stable High** |  |  |  |
| **Maternal age** (years) |  |  |  |
| Spline 1* | **0.69 (0.49, 0.97)** | **0.69 (0.49, 0.98)** | 0.836 |
| Spline 2** | 1.05 (0.95, 1.16) | 1.06 (0.94, 1.2) | 0.793 |
| **Maternal education** |  |  |  |
| Medium (ref.: Low) | 1.47 (0.39, 5.55) | 0.78 (0.1, 5.87) | 0.507 |
| High (ref.: Low) | 1.17 (0.25, 5.46) | 2.78 (0.28, 27.67) | 0.495 |
| **Paternal education** |  |  |  |
| Medium (ref.: Low) | 0.68 (0.29, 1.63) | 0.75 (0.22, 2.51) | 0.770 |
| High (ref.: Low) | 0.98 (0.29, 3.28) | 0.28 (0.04, 1.98) | 0.487 |
| **Maternal working status** (pregnancy) |  |  |  |
| Working (ref.: Unemployed) | 2 (0.62, 6.45) | 1.25 (0.25, 6.2) | 0.489 |
| **Household income** (tertiles) |  |  |  |
| 2^nd^ Tertile (ref.: 1^st^) | 0.88 (0.31, 2.53) | 1 (0.17, 5.9) | 0.816 |
| 3^rd^ Tertile (ref.: 1^st^) | 0.52 (0.14, 1.94) | 0.37 (0.04, 3.72) | 0.925 |
| **Breastfeeding duration** (months) | 0.95 (0.86, 1.05) | 0.99 (0.85, 1.15) | 0.567 |

Note: Reference: Stable Low trajectory.

Bold font indicates p < 0.05.

^#^The p-interactions values were obtained from models including a multiplicative interaction term between risk factor and sex (male, female).

*Spline 1: maternal age≤30 for internalizing symptoms and ≤25 for externalizing and ADHD symptoms.

**Spline 2: maternal age>30 for internalizing symptoms and >25 for externalizing and ADHD symptoms.
